# A Proof-of-Concept Study: Reference Transcriptome Choice as a Hidden Analytical Variable in Breast Cancer RNA-Seq Analysis

**DOI:** 10.3390/ijms27219398

**Published:** 2026-10-22

**Authors:** Paweł Zieliński, Magdalena Frąszczak, Bartosz Czech, Paula Dobosz, Kinga Adamiak, Patryk Mierzejewski, Rafał Stępień, Magda Mielczarek

**Affiliations:** 1 Pathomorphology and Clinical Immunology, Poznan University of Medical Sciences, 49 Przybyszewskiego St., 60-356 Poznań, Poland; 2 Department of Genetics, Wroclaw University of Environmental and Life Sciences, 7 Kożuchowska St., 51-631 Wroclaw, Poland

**Keywords:** breast cancer, genomic annotations, NGS, transcript expression profiling, RNA-seq

## Abstract

Reference genome and transcriptome are fundamental components of RNA-seq analysis and can influence transcript quantification and downstream differential expression results. Although GRCh38 is the current human reference build, GRCh37 remains in use in research and clinical bioinformatics workflows. The extent to which reference transcriptome choice affects transcriptomic inference in breast cancer remains insufficiently characterized. We performed a comparative analysis of publicly available RNA-seq data comprising two MCF7 breast cancer cell-line samples and two healthy breast tissue samples from the European Nucleotide Archive (PRJEB4829). Identical preprocessing, transcript quantification, and differential expression workflows were applied using GRCh37- and GRCh38-based reference transcriptomes. Reference transcriptome-dependent variability was further examined by comparing the same samples quantified under the two reference transcriptomes. Concordance of differentially expressed genes and pathway enrichment was assessed, and sensitivity to reference transcriptome choice was evaluated for genes included in the MammaPrint and Naderi expression signatures and selected hereditary cancer gene panels. The MCF7-versus-normal-tissue comparison identified 15,950 differentially expressed genes using GRCh37 and 16,348 using GRCh38, with 13,899 genes shared between the two analyses. The most significantly enriched Hallmark and KEGG pathways were also concordant between annotations. Principal component analysis showed that the dominant pattern of variation in the MCF7-versus-normal-tissue comparison was associated with biological status. In contrast, when the same MCF7 samples were quantified using different reference transcriptomes, reference transcriptome choice represented a major source of variation. Expression of genes comprising the evaluated prognostic signatures and hereditary cancer panels showed no significant differences in median expression between reference transcriptomes. However, individual cancer-relevant genes, including *PTEN*, *RAD51D*, *NANOG*, and *SOX11*, demonstrated reference transcriptome-dependent expression differences. In this proof-of-concept analysis, reference transcriptome choice produced measurable differences in RNA-seq quantification and differential expression, while broad biological patterns and the aggregate expression of established gene signatures were relatively stable. Reference transcriptome-dependent variability became particularly apparent when biological heterogeneity was limited, suggesting that reference transcriptome choice can act as a hidden analytical variable in transcriptomic studies. These findings support explicit reporting and version control of reference annotations and highlight the need for validation in larger, independently replicated breast cancer cohorts before drawing clinical conclusions.

## 1. Introduction

Breast cancer is the most frequently diagnosed cancer type in women and is the second most common cause of cancer death among women worldwide [1,2]. It affects one in seven women during their lifetime [3] and accounts for 23% of all diagnosed cancers and 14% of cancer deaths [4]. Although incidence rates vary significantly across different regions, breast cancer remains a critical health concern, with projections estimating nearly 3 million new cases by 2040. This highlights the persistent and growing challenge posed by breast cancer on a global scale. Due to huge disease complexity, many different classifications for breast cancer have been proposed over the years and they may be based on the cancer stage, visual examination of abnormal cells under a microscope, the presence or absence of certain receptors, molecular subtype or specific gene expression [5]. High-throughput sequencing methods have brought breast cancer research into a new era by allowing for whole genome/transcriptome exploration and personalised medicine [6,7]. It offers tools that enable the pathologist to combine morphological analyses with molecular alterations in challenging diagnostic situations [8]. By studying transcriptome-level genetic information in the cell, it is possible to determine which genes/transcripts are expressed on different levels in cancer-affected and normal tissues. Interpreting those results can reveal the background of cancer development and give a better view of which treatment should be used. In fact, a gene expression profiling is a key to understanding breast cancer and individualizing its treatment—even if it only remains a part of a bigger concept named multi-omics, which we are only at the very beginning of understanding. Clinical oncologists are eager to use the results of these studies in their day-to-day practices, therefore understanding the technique itself is also essential [9,10].

The choice of reference genome/transcriptome is pivotal for the interpretation of gene expression data in breast cancer research [11]. An appropriate reference sequence supports reliable gene expression quantification and the identification of differentially expressed genes (DEGs) and alternative splicing events, thereby contributing to the molecular characterization of breast cancer and potentially informing treatment-related research [11,12]. Conversely, outdated or incomplete reference can lead to misinterpretations, potentially overlooking critical biomarkers or therapeutic targets. Modern RNA-seq technologies rely heavily on reference genome/transcriptome to quantify gene expression accurately, facilitating the discovery of novel transcripts and non-coding RNAs [13]. As transcript databases evolve, reanalysis of existing datasets often reveals new insights, underscoring the dynamic nature of genomic research. Therefore, genomic and transcriptomic reference sequences are not merely supplementary resources but foundational elements that directly influence the outcomes and translational impact of breast cancer gene expression studies [11,12].

In this study, we analysed publicly available transcriptomic data from healthy breast tissue and MCF-7 breast cancer cells obtained from the European Nucleotide Archive (ENA; https://www.ebi.ac.uk/ena accessed on 20 April 2021) under project accession PRJEB4829. MCF-7 is one of the most widely used human breast cancer cell lines and has been extensively employed as an in vitro model for investigating breast cancer biology, progression, and therapeutic responses [4]. Its established use in breast cancer research therefore provides a well-characterized experimental model for evaluating reference transcriptome-dependent differences in RNA-seq analyses. To assess the potential impact of the choice of reference transcriptome on RNA-seq results, we analysed the same datasets using GRCh37-based and GRCh38-based reference transcriptomes obtained from Ensembl [14]: GRCh38, representing the more recent reference genome build, and GRCh37, the preceding build that remains relevant to human genomic studies and continues to be used in research and clinical settings [15,16,17]. Although the use of an up-to-date reference annotation is generally preferred for contemporary transcriptomic analyses, continued use of previous genome builds may affect the reproducibility and comparability of results generated across different bioinformatic workflows. We therefore investigated whether the choice between GRCh37- and GRCh38-based reference transcriptomes could introduce systematic differences in transcript quantification and differential gene expression, as well as affect the expression patterns of selected cancer-related gene sets.

### Aim of the Study

The aim of this proof-of-concept study was to investigate whether the choice of reference transcriptome represents a hidden analytical variable in RNA-seq studies by comparing GRCh37- and GRCh38-based transcript quantification and differential gene expression in a breast cancer model. We further evaluated whether reference transcriptome-dependent variability affects the reproducibility of selected biologically and clinically relevant gene sets and whether its contribution to transcriptomic variation increases when biological heterogeneity is limited.

**Figure 1:**
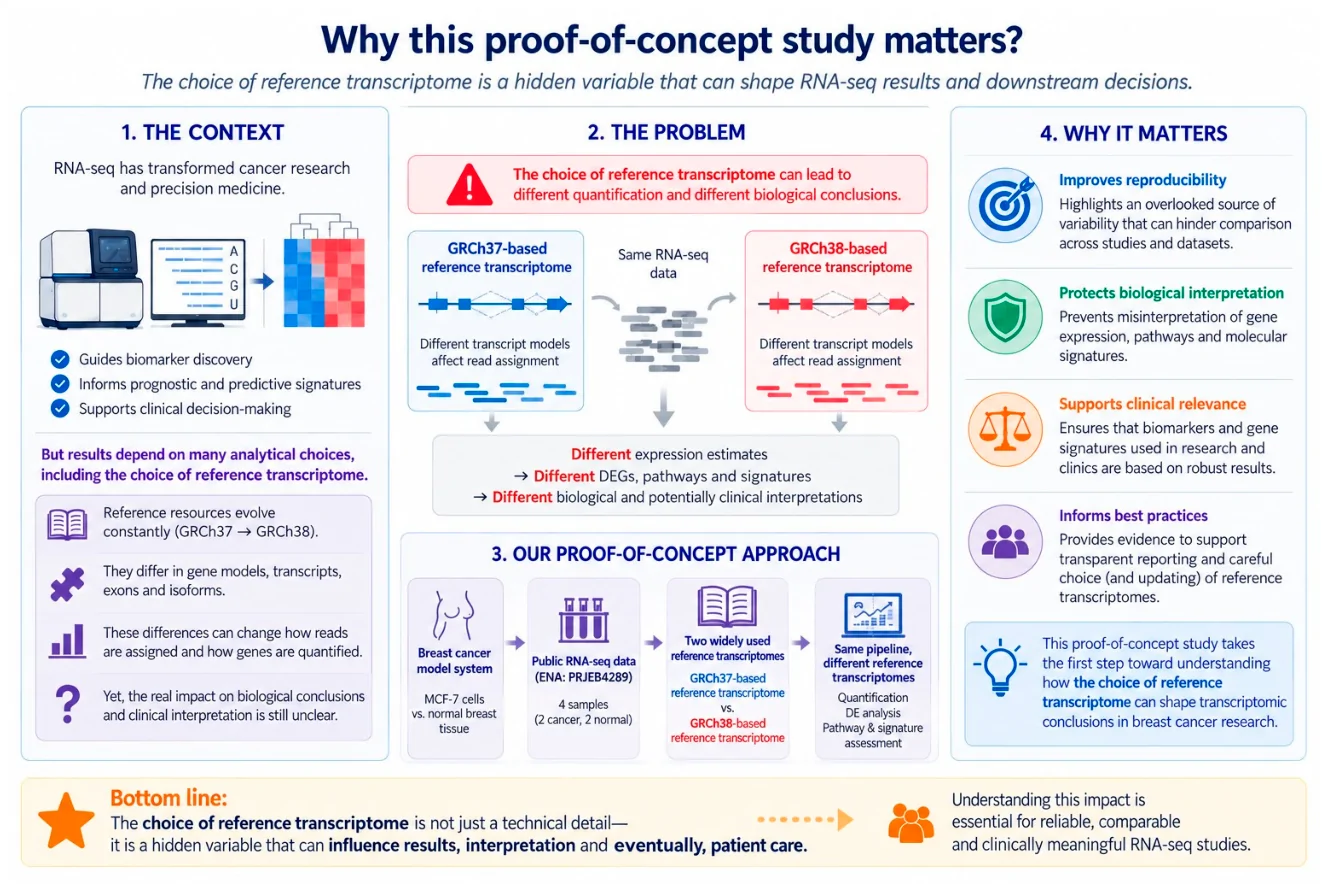
Rationale for the proof-of-concept study, illustrating how reference transcriptome can influence RNA-seq quantification and downstream biological interpretation of breast cancer transcriptomic analyses.

Our intention is not to demonstrate that GRCh37 and GRCh38 differ per se, but to assess how these differences are reflected across multiple levels of a transcriptomic analysis, from read quantification and differential gene expression to functional interpretation and the assessment of the evaluation of well-established breast cancer-related genes included in selected gene panels. In this context, the study focuses on the practical consequences of reference transcriptome choice for downstream biological interpretation. The importance of this concept is depicted in Figure 1 below.

Interestingly, our study was not limited to conventional case–control comparisons but also included case–case and control–control comparisons across the two reference annotations. This design allowed us to examine transcriptome-dependent variability within the same biological condition, thereby limiting the contribution of biological differences between the analysed sample types. Consequently, these approaches provided a more direct assessment of the contribution of the choice of reference transcriptome to the observed variation in gene expression and enabled us to determine whether this effect becomes more apparent when biological heterogeneity is limited.

## 2. Results

### 2.1. Bioinformatic Workflows

Quality-based data trimming was performed and resulted in dropping out from 1.92 to 3.09% of reads per sample. Transcript expression was quantified using the trimmed RNA-seq reads against GRCh37-based and GRCh38-based reference transcriptomes, which differ in transcript content, structure, and sequence, including the number of represented transcripts. The total number of transcripts for GRCh38 was 248,592 (190,522 cDNA and 58,070 ncRNA sequences) and for GRCh37—215,170 (180,253 cDNA and 34,917 ncRNA) what changed the result of expression quantification. Maximum TPM (Transcripts Per Million) values for GRCh38 and GRCh37 annotations were 27,098.4 and 21,046.3 for normal and 19,085.3 and 19,378 for cancer tissues. Both reference transcriptomes identified the same transcripts as the most highly expressed in both normal (ENST00000390237) and cancer (ENST00000361227) samples.

### 2.2. Case–Control

**Figure 2:**
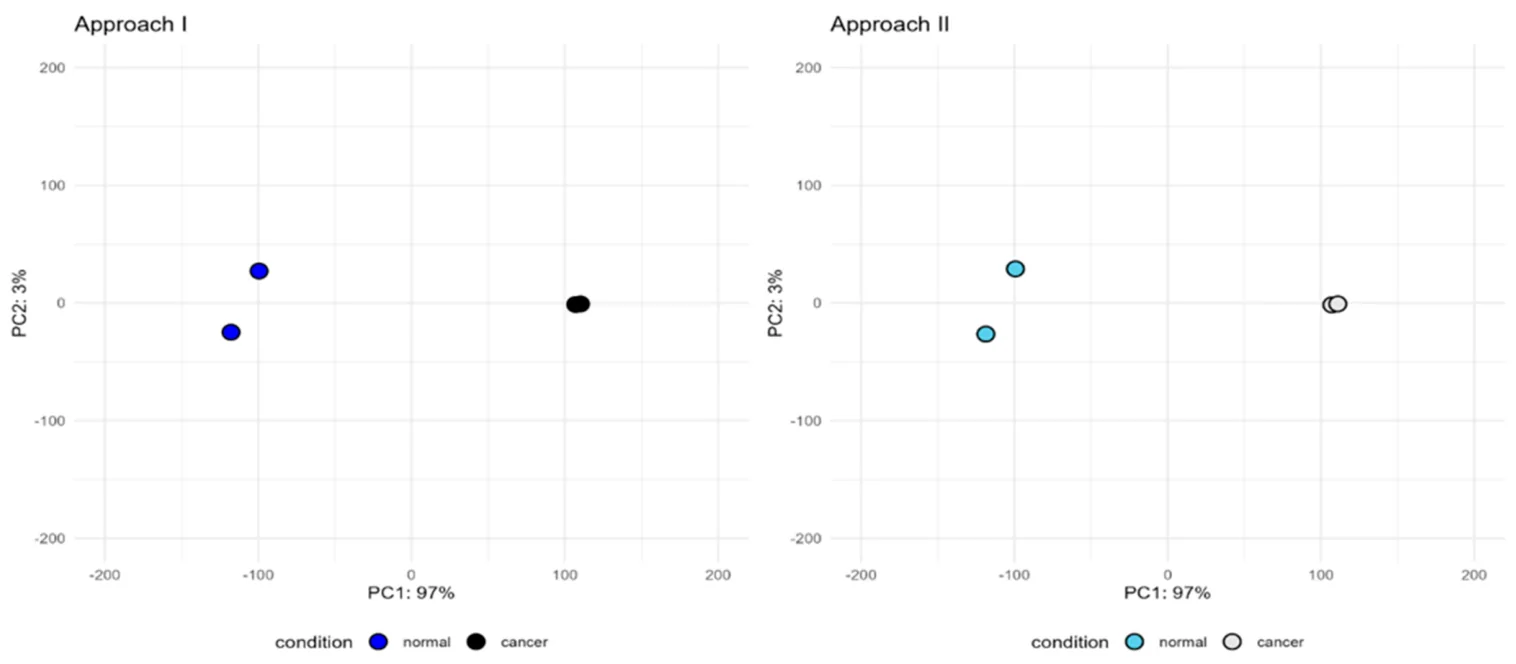
Principal Component Analysis (PCA) demonstrating the clustering of cancer and normal samples in both Approach I (**Left**) and Approach II (**Right**).

In the Principal Component Analysis (PCA) including cancer and normal samples, PC1 accounted for approximately 97% of the total variance and separated the samples according to biological condition in both Approaches I (Figure 2(left)) and II (Figure 2(right)).

**Figure 3:**
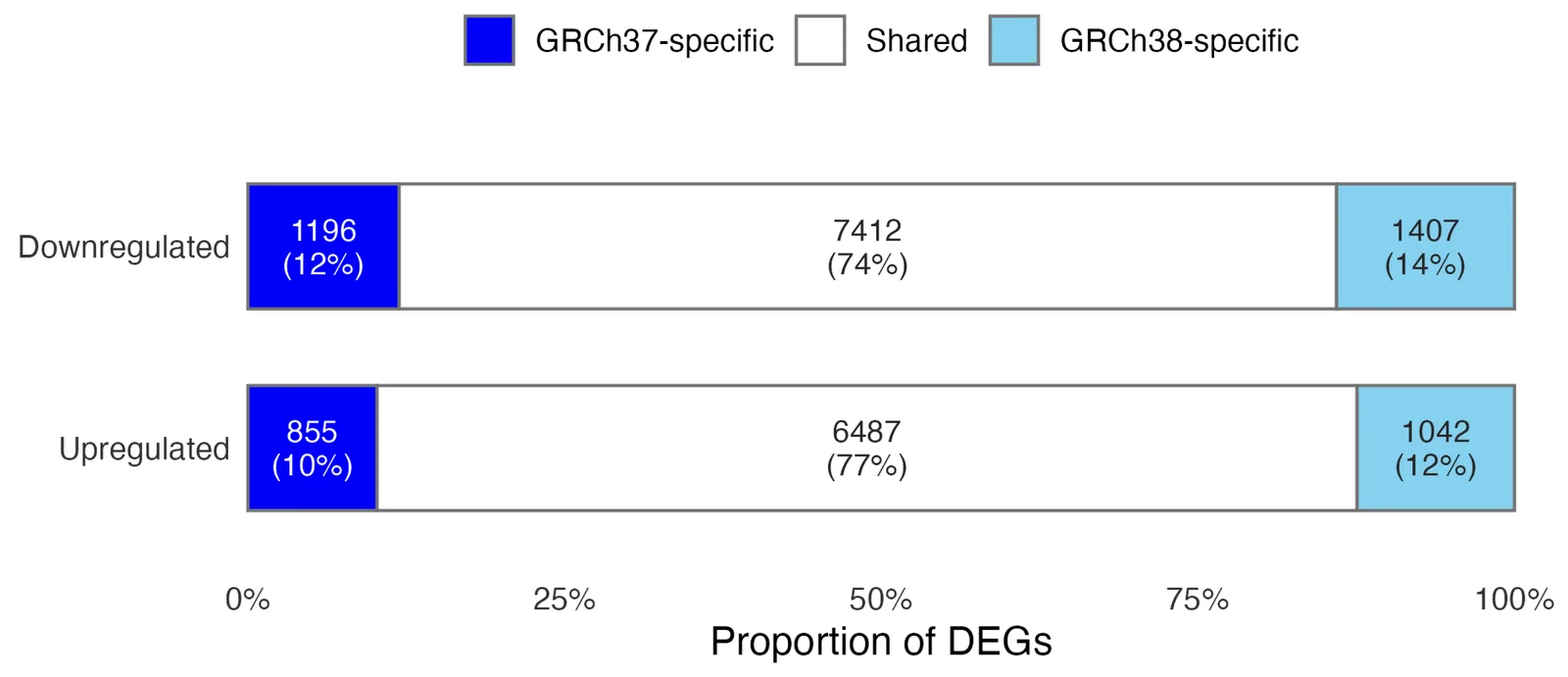
Overlap of differentially expressed genes identified in cancer versus normal tissue comparisons across both approaches, comprising 6487 shared up- (**Bottom**) and 7412 downregulated transcripts (**Top**).

A total of 15,950 DEGs were identified for GRCh37 (Approach I), comprising 7342 up- and 8608 downregulated genes. For the GRCh38 (Approach II) annotations, 16,348 DEGs were found (7529 up- and 8819 downregulated). The overlap between cancer and normal tissue comparisons for both approaches included 13,899 genes (6487 up- and 7412 downregulated) (Figure 3).

**Figure 4:**
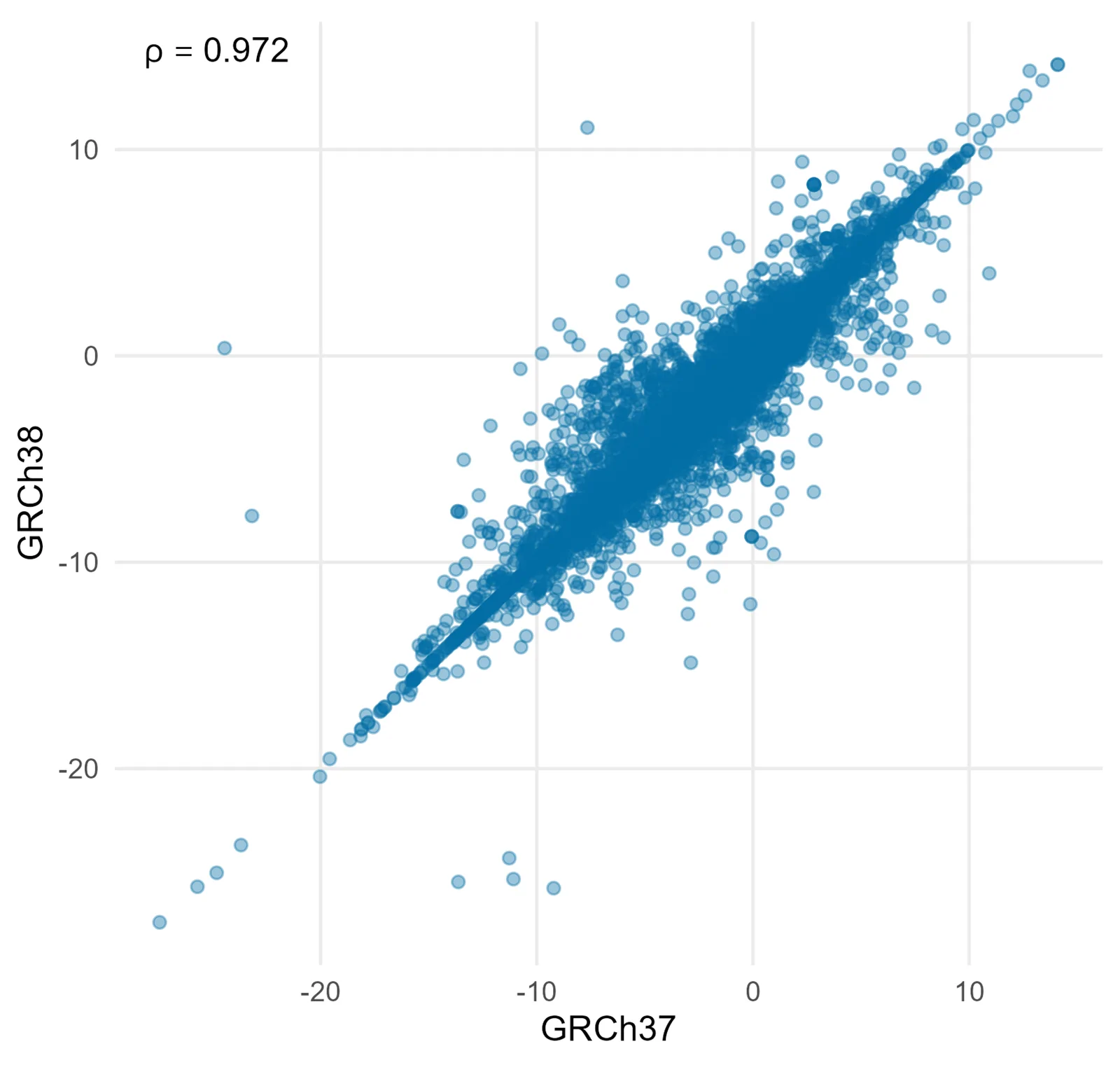
Correlation of log2FC estimates obtained using GRCh37-based (Approach I) and GRCh38-based (Approach II) reference transcriptomes. Each point represents an individual gene. The Spearman correlation coefficient (ρ = 0.972) indicates a strong positive correlation between log2FC estimates obtained using the two reference transcriptomes.

**Figure 5:**
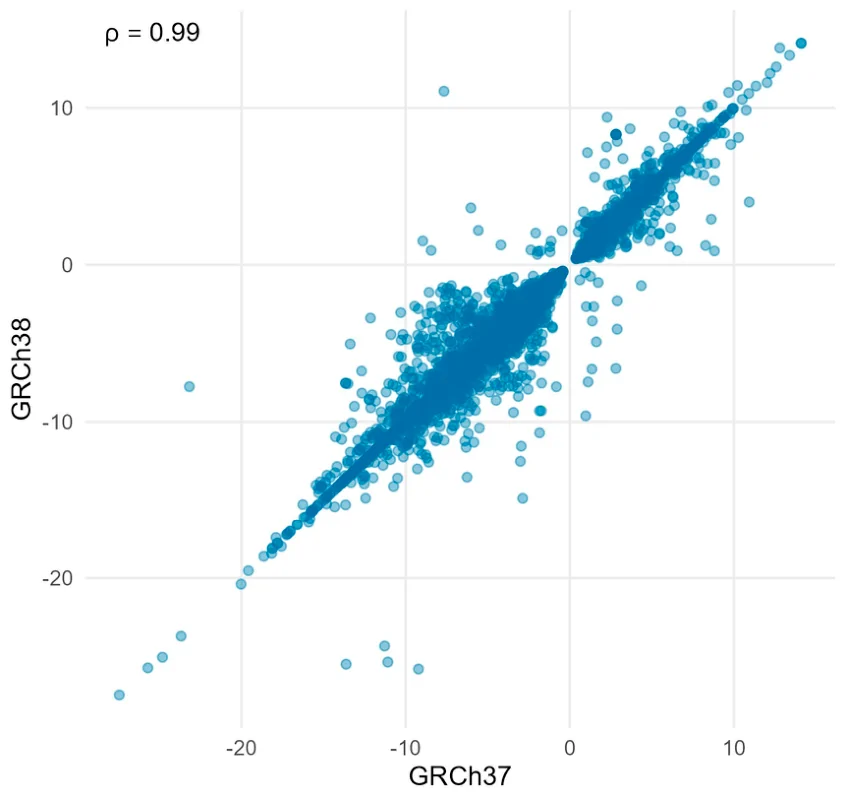
Correlation of log2FC values for differentially expressed genes identified using both reference transcriptomes. The plot shows the relationship between log2FC estimates for genes identified as statistically significant DEGs using both the GRCh37-based and GRCh38-based reference transcriptomes. Each point represents an individual gene. The Spearman correlation coefficient (ρ = 0.99) indicates a strong positive correlation between log2FC estimates obtained using the two reference transcriptomes.

The agreement between the Approach I and Approach II was further examined using Spearman correlation. Correlations between log2FC estimates obtained using the two reference transcriptomes were calculated for all genes (Figure 4) and, separately, for genes identified as statistically significant DEGs in both analyses (Figure 5). In both comparisons, the *p*-values in the Spearman correlation tests converged asymptotically to zero.

**Figure 6:**
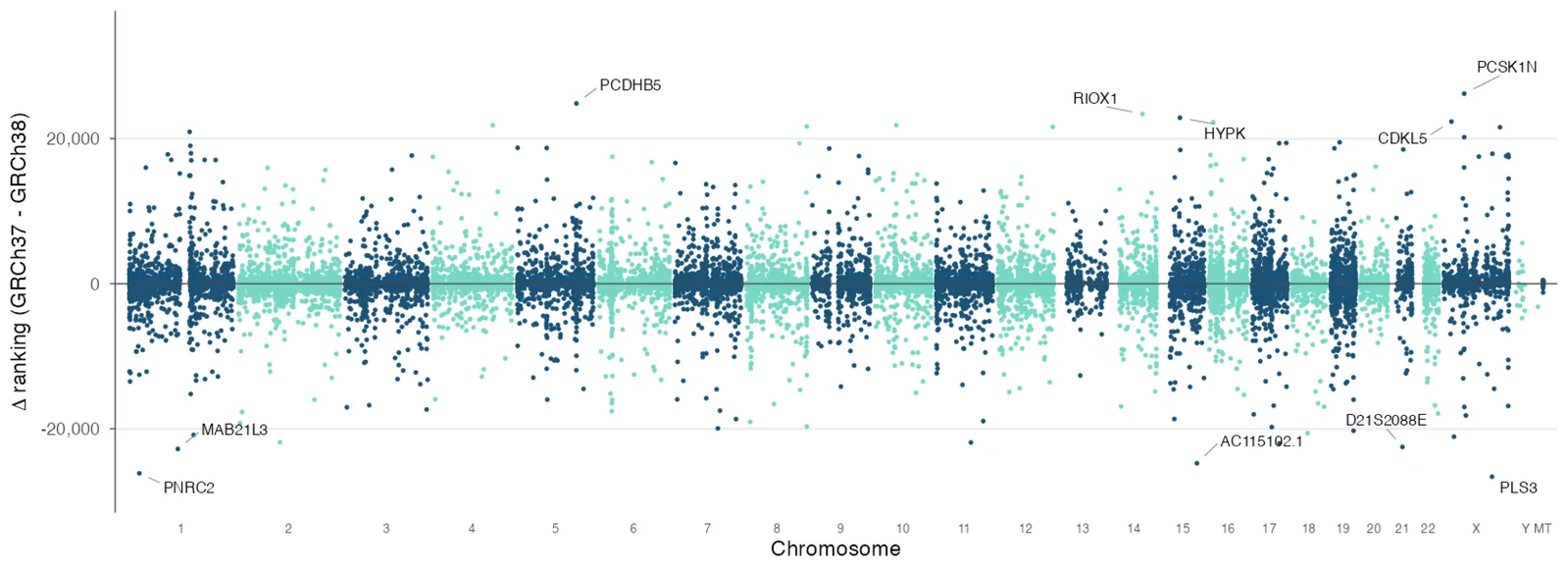
Gene-ranking concordance between Approaches I and II. Differences in gene ranks between two approaches are shown across chromosomes. Each point represents an individual gene. The horizontal line at zero indicates identical gene ranks in both approaches. Positive values indicate a higher rank in the GRCh38-based analysis, whereas negative values indicate a higher rank in the GRCh37-based analysis. Genes showing the largest rank differences in each direction are labelled.

**Figure 7:**
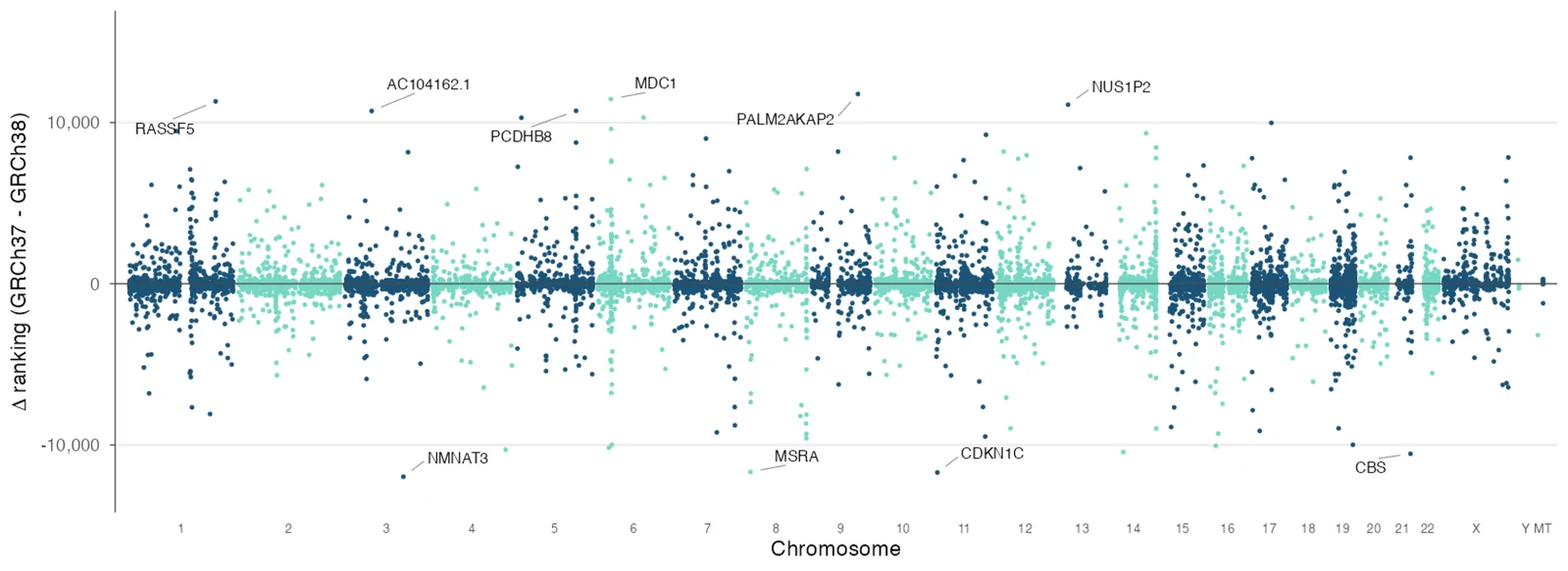
Gene-ranking concordance between Approaches I and II for statistically significant DEGs. Differences in gene ranks between the two approaches are shown across chromosomes for genes identified as statistically significant in both analyses. Each point represents an individual gene. The horizontal line at zero indicates identical gene ranks in both approaches. Positive values indicate a higher rank in the GRCh38-based analysis, whereas negative values indicate a higher rank in the GRCh37-based analysis. Genes showing the largest rank differences in each direction are labelled.

Concordance between the GRCh37-based and GRCh38-based analyses was further evaluated using two complementary comparisons. Gene ranks of all genes were compared between Approach I and Approach II (Figure 6). Differences in gene ranking were observed across all chromosomes, although the majority of genes showed relatively small shifts between the two reference transcriptomes Among ten genes with the largest absolute rank differences, PCDHB5, RIOX1, HYPK, CDKL5, and PCSK1N showed positive rank differences, whereas MAB21L3, PNRC2, AC115102.1, D21S2088E, and PLS3 showed negative rank differences. Additionally, gene-ranking concordance was assessed separately for genes identified as statistically significant in both approaches (Figure 7). Among ten genes with the largest absolute rank differences in this subset, RASSF5, AC104162.1, PCDHB8, MDC1, PALM2-AKAP2, and NUS1P2 showed positive rank differences, whereas NMNAT3, MSRA, CDKN1C and CBS showed negative rank differences.

**Figure 8:**
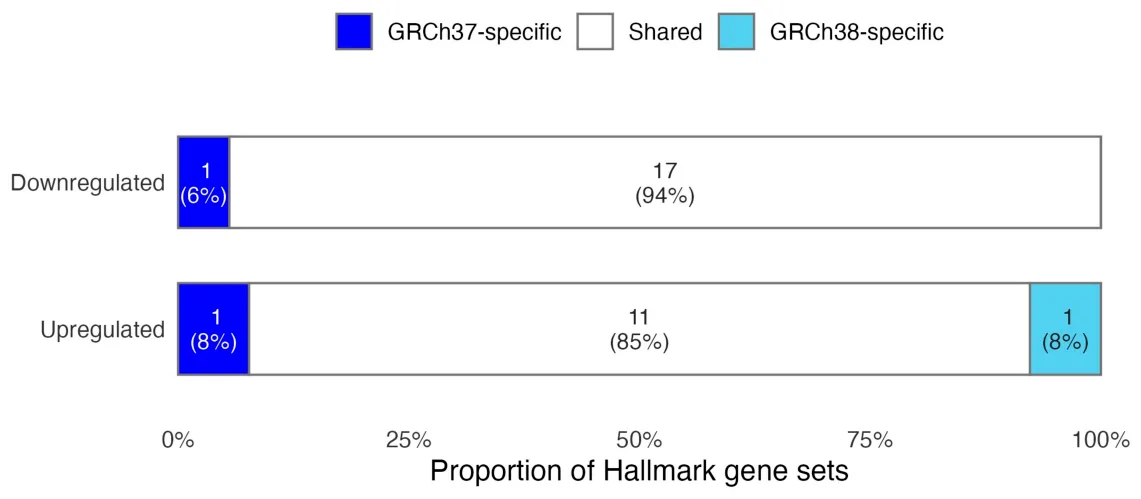
Comparison of Molecular Signatures Database (MSigDB) Hallmark enrichment results between Approach I and Approach II. Enrichment analyses were performed separately for (**Bottom**) up- and (**Top**) downregulated gene sets.

**Figure 9:**
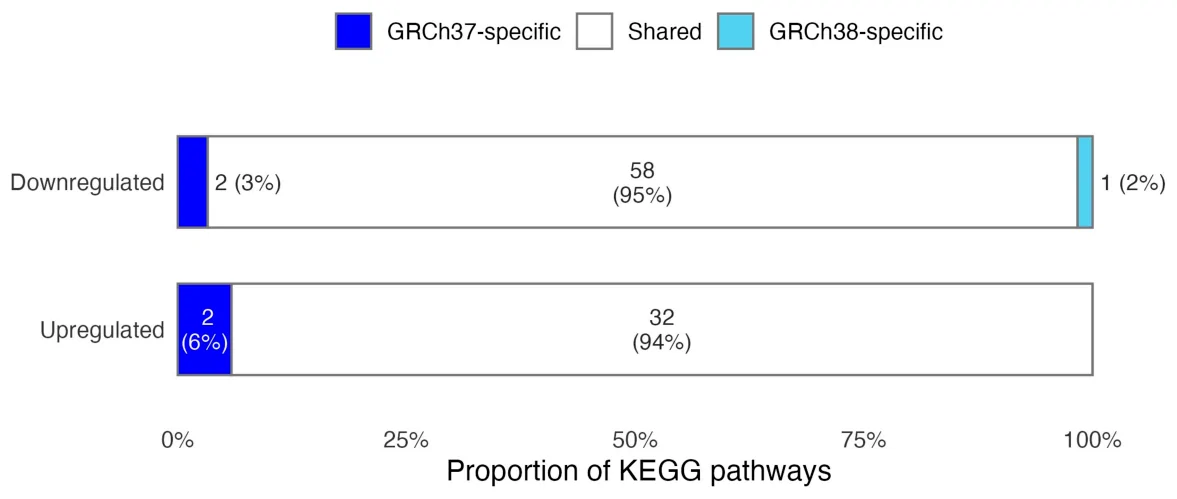
Comparison of Kyoto Encyclopedia of Genes and Genomes (KEGG) enrichment results between Approach I and Approach II. Enrichment analyses were performed separately for (**Bottom**) up- and (**Top**) downregulated gene sets.

The Molecular Signatures Database (MSigDB) Hallmark gene set [18] enrichment analysis revealed largely consistent results between the two analytical strategies (Figure 8). Both approaches identified the same statistically significant gene signatures, with the exception of three pathways: two uniquely enriched in Approach I (HALLMARK_REACTIVE_OXYGEN_SPECIES_PATHWAY, HALLMARK_INTERFERON_ALPHA_RESPONSE) and one uniquely enriched in Approach II (HALLMARK_ESTROGEN_RESPONSE_LATE). For Kyoto Encyclopedia of Genes and Genomes (KEGG) [19] still, the vast majority (90) of significantly enriched pathways were common between Approaches I and II (Figure 9). Private KEGG pathways for Approach I included four pathways (cellular senescence, viral carcinogenesis, vascular smooth muscle contraction, C-type lectin receptor signalling pathway), while for Approach II one pathway (neuroactive ligand-receptor interaction). The complete list of significantly enriched pathways is provided in Supplementary Materials S1 (Hallmark) and S2 (KEGG).

### 2.3. Case-Case and Control-Control

**Figure 10:**
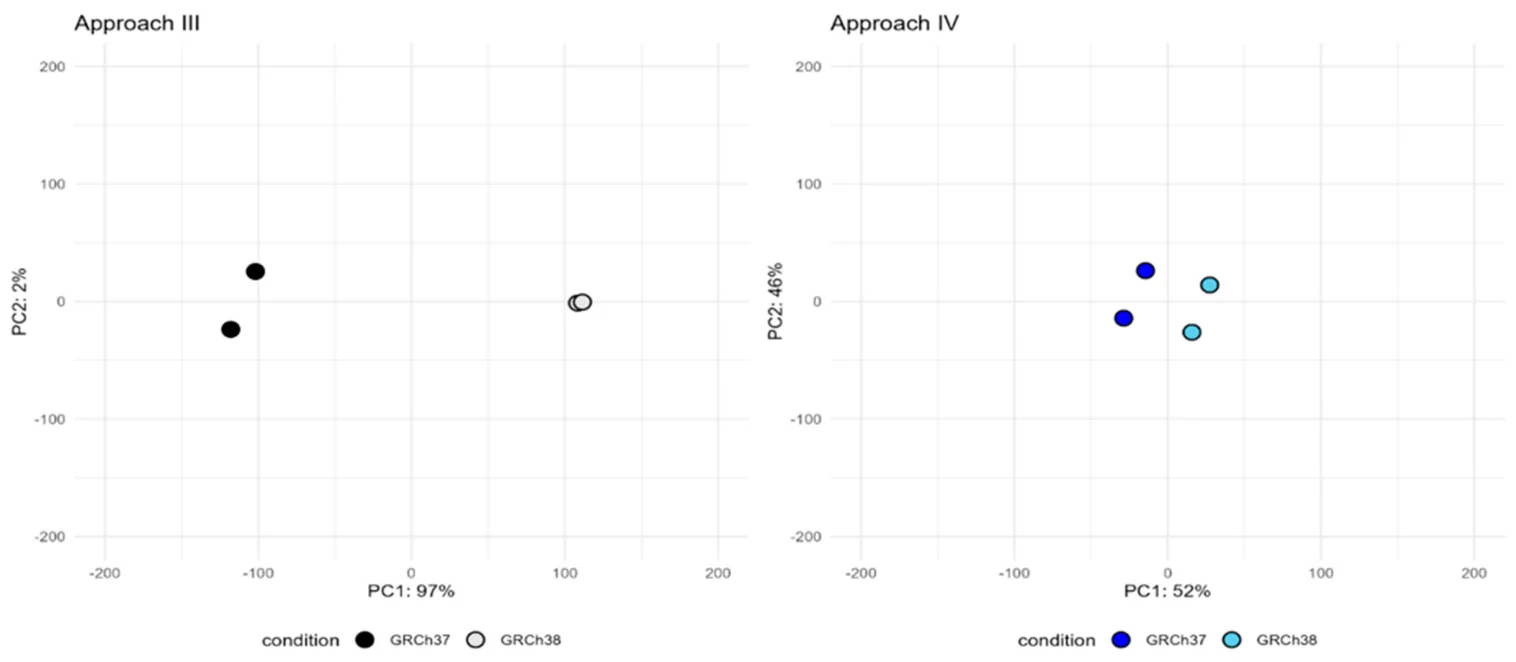
Principal Component Analysis (PCA) demonstrating the clustering of cancer (**Left**) and normal (**Right**) samples quantified on different annotation versions.

Interestingly, the PCA plot for Approach III, in which only cancer samples were compared, showed a clear separation associated with the reference transcriptome used for quantification (Figure 10(left)). In Approach IV, which included only healthy samples, PC1 accounted for 58% of the variance and primarily separated the biological samples, whereas separation associated with the choice of reference transcriptome was observed along PC2, which accounted for 40% of the variance (Figure 10(right)).

**Figure 11:**
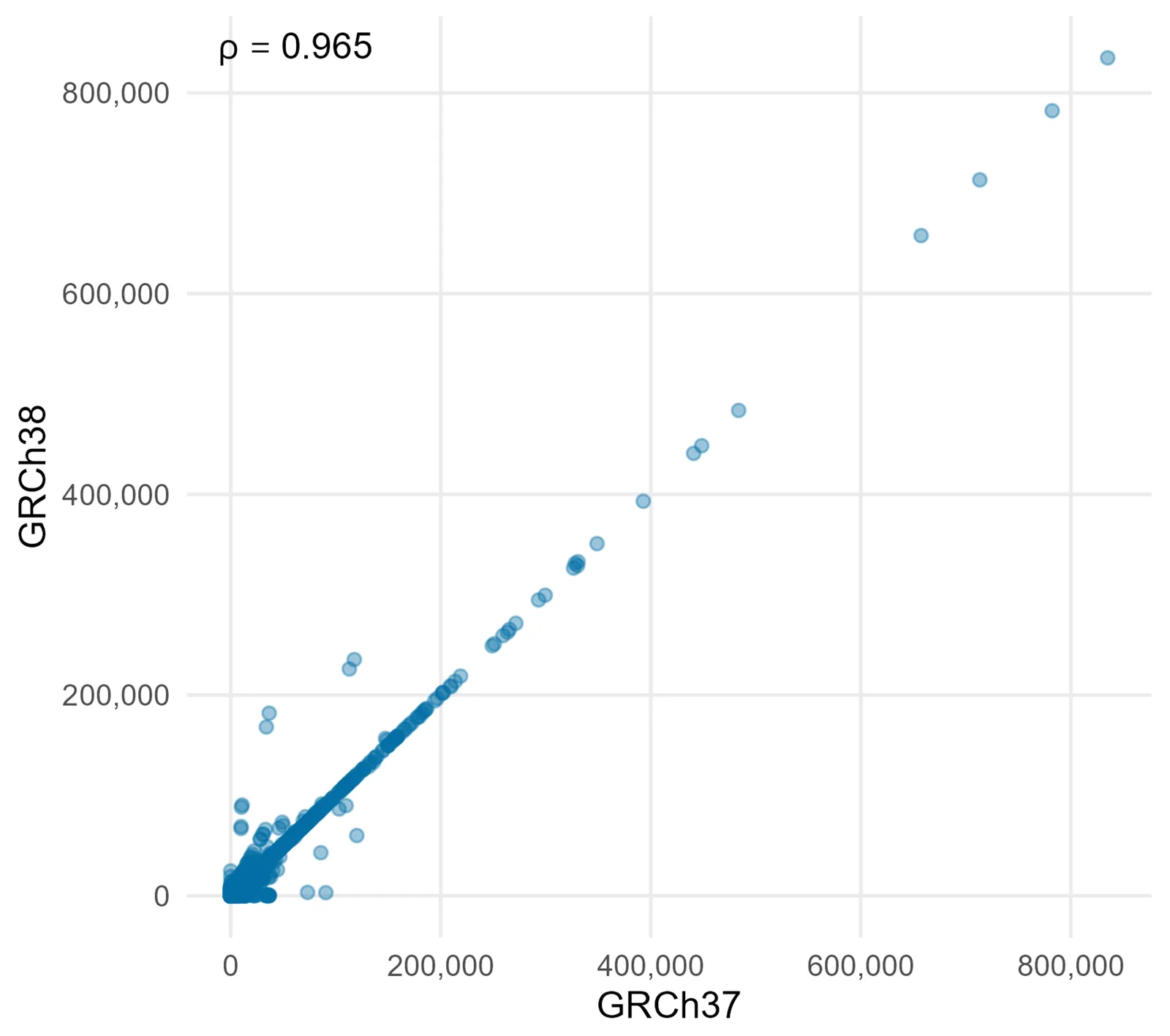
Correlation of gene expression levels between GRCh37- and GRCh38-based reference transcriptomes (Approach III). Each point represents an individual gene. The Spearman correlation coefficient (ρ = 0.965) indicates a strong positive correlation between gene expression estimates obtained using the two reference transcriptomes.

A strong positive correlation in gene expression levels was observed between the GRCh37- and GRCh38-based reference frameworks (Spearman’s ρ = 0.972; Figure 11), indicating a high overall concordance of gene-level expression estimates between the two approaches.

### 2.4. Assessment of Genes of Clinical Interest Across Reference Transcriptomes

**Figure 12:**
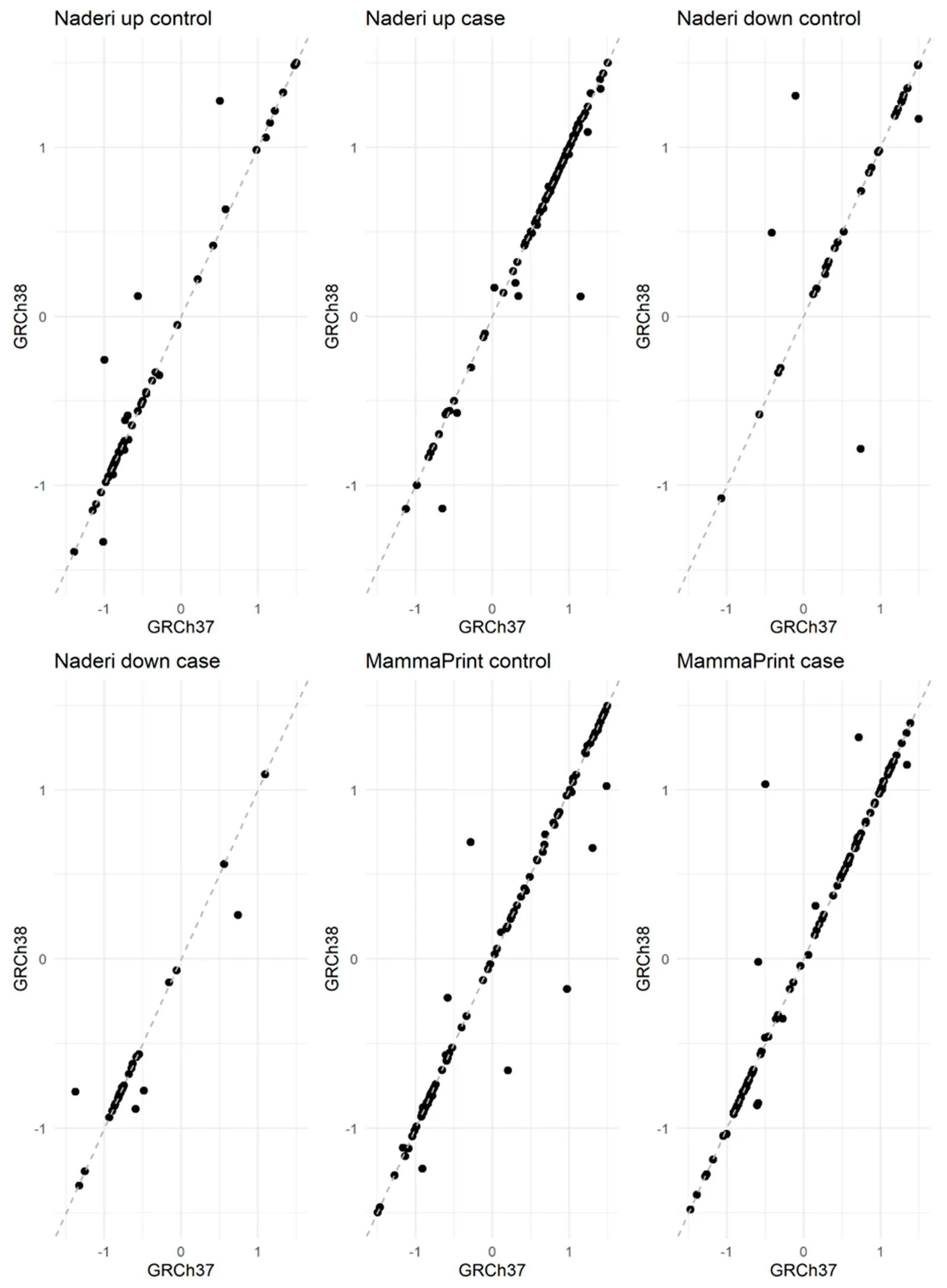
The expression differences between both annotations for all MammaPrint and Naderi signatures subsets. The expression differences between genes quantified on GRCh37 and GRCh38 located on the trend line (dashed line) are close to or equal to zero.

**Figure 13:**
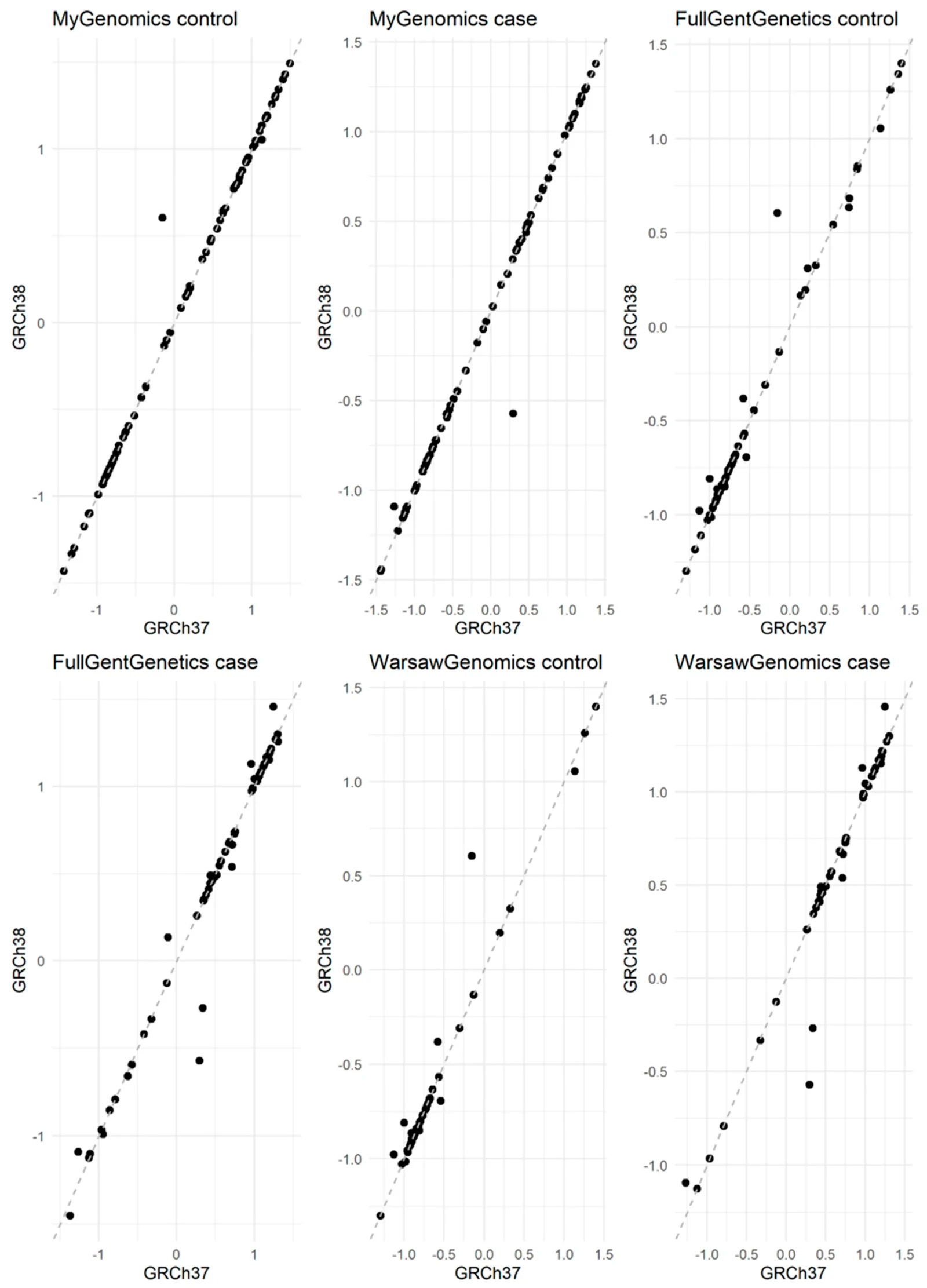
The expression differences between both annotations for all gene panels. The expression differences between genes quantified on GRCh37 and GRCh38 located on the trend line (dashed line) are close to or equal to zero.

The Wilcoxon signed-rank test was conducted on genes determined in gene expression signatures panels such as (1) MammaPrint [20,21,22] and (2) Naderi [23] for (a) up- and (b) downregulated genes. Moreover, the test was also performed for genes from gene panels devoted to determining the DNA variation and they were (3) WarsawGenomics (www.warsawgenomics.pl), (4) FullGentGenetics (www.fulgentgenetics.com) and (5) MyGenomics (www.mygenomics.com). For each subset, the statistical testing indicated no significant differences in median gene expression levels between those quantified using the GRCh37 and GRCh38-based transcriptoms. For Mammaprint, the obtained *p*-values were as follows: *p* = 0.73 for the case-case comparison and *p* = 0.25 for the control-control comparison. For Naderi’s signatures, the *p*-values were *p* = 0.14 for the case-case and *p* = 0.36 for the control-control comparison (upregulated genes) as well as *p* = 0.83 for the case-case and *p* = 0.93 for the control-control comparison (downregulated genes), Results based on gene panels were as follows: for WarsawGenomics *p* = 0.97 (case-case) and *p* = 0.68 (control-control), for FullGentGenetics *p* = 0.52 and *p* = 0.24, and for MyGenomics *p* = 0.99 and *p* = 0.92. Figure 12 and Figure 13 show the expression differences between both annotations for all gene subsets. The expression differences between genes quantified on GRCh37 and GRCh38 located on the trend line are close to or equal to zero. Genes for which expression differs are located to the right or left of the trend line. Statistically significant outliers, determined using the percentile bootstrap confidence intervals, are listed in Table 1.

## 3. Discussion

Breast cancer is increasingly viewed as a highly heterogenous disease; its diagnosis involves clinical evaluation through breast examination and medical history, followed by imaging techniques such as mammography, ultrasound, and MRI for high-risk patients [24,25]. Subsequent biopsy methods, including core needle biopsy, fine needle aspiration, and excisional biopsy, confirm the diagnosis and enable pathological assessment of cancer type and receptor status (ER, PR, HER2). Increasingly, advanced genetic testing further supports diagnosis and personalised treatment selection. Treatment may include surgery and radiotherapy, as well as hormone therapy (e.g., tamoxifen, aromatase inhibitors), targeted therapies (HER2, CDK4/6 and PARP inhibitors), and immunotherapy (e.g., pembrolizumab for PD-L1-positive cancers) [24,26]. Emerging advancements include genomic profiling to personalize treatment based on both, tumour-specific genetic mutations and gene expression [24,27,28]. Traditional chemotherapy (neoadjuvant or adjuvant) remains important for aggressive, unresectable, or large tumours, while multi-omics increasingly supports personalised treatment selection and predictive modelling. Ongoing clinical trials focus on novel targeted therapies, including for triple-negative breast cancer, and strategies to prevent and treat metastatic disease [29]. The tumour-agnostic approach is increasingly important in precision oncology. For example, HER2-targeting antibodies, initially approved for specific cancers, have recently shown efficacy across multiple solid tumours, including gynaecological cancers with substantial unmet therapeutic needs [30,31]. This tumour-agnostic approach was pioneered by larotrectinib, the first FDA-approved tissue-agnostic therapy targeting NTRK gene fusions in adult and paediatric patients with advanced solid tumours lacking satisfactory treatment alternatives [31,32].

However, in order to use the latest scientific achievements in the clinic, we need to determine good biomarkers, as well as best possible tools and good laboratory practice rules including in bioinformatics. Gene annotations are foundational to RNA-seq analyses, influencing gene quantification, differential expression detection, and ultimately clinical interpretations. The choice between genome builds, such as GRCh37 and GRCh38, introduces variability due to differences in gene models, exon-intron structures, and transcript definitions. This variability can significantly affect diagnostic outcomes in breast cancer studies [33]. It is crucial for all of us to apprehend that modern molecular diagnostic units rely more on bioinformatics than the wet lab itself. Thus, in this work, we focused on the choice of reference transcriptome, having noticed the differences between laboratories.

Reference transcriptome choice acts as a hidden technical variable in RNA-seq studies, with limited impact on established clinical signatures but substantial influence on exploratory gene discovery analyses. Our analysis revealed significant discrepancies in both the number and identity of differentially expressed genes (DEGs). These differences are attributable to updates in gene models, including the addition or removal of transcripts, redefinition of exon-intron boundaries, and the incorporation of novel splice variants. Such modifications can substantially influence the quantification of gene expression levels, leading to divergent biological interpretations [34].

Reference transcriptome is a fundamental but frequently underappreciated component of RNA-seq analysis. Differences between genome builds can alter transcript definitions, exon boundaries, isoform representation, and the assignment of sequencing reads, thereby influencing downstream estimates of transcript abundance and differential expression [33,34]. In the present study, we systematically compared two commonly used human reference configurations, GRCh37 and GRCh38, while keeping the sequencing data and the principal bioinformatic workflow unchanged. Our results demonstrate that transcriptome choice can produce measurable differences in transcript quantification and in the set of genes identified as differentially expressed, despite the underlying sequencing libraries being identical. These findings support the concept that reference framework should be considered an explicit analytical variable rather than a purely technical detail of an RNA-seq workflow.

The magnitude and interpretation of transcriptome-dependent variability, however, depended strongly on the biological comparison being performed. In the MCF-7-versus-normal-tissue comparison, the two reference transcriptoms produced broadly consistent gene expression profiles. GRCh37 and GRCh38 identified 15,950 and 16,348 differentially expressed genes, respectively, with 13,899 genes shared between the two analyses. Similarly, most significantly enriched Hallmark and KEGG pathways were common to both annotation versions.

PCA showed that PC1 accounted for approximately 97% of the observed variance in both analytical approaches and clearly separated MCF-7 cells from normal breast tissue. This indicates that the distinction between the two biological conditions corresponded to the dominant pattern of variation in the analysed dataset. Importantly, this broad concordance did not imply complete equivalence at the gene level, as a subset of genes and pathways showed differences associated with the choice of reference transcriptome. The dominant pattern of variation was associated with the separation between cancer and normal tissue samples, what is consistent with studies demonstrating that tumour and normal tissues can exhibit markedly distinct global gene expression profiles in PCA [35]. Large pan-cancer transcriptomic datasets from The Cancer Genome Atlas (TCGA) also reveal clear separation between tumour and normal tissue in PCA, indicating that biological status is strongly associated with major patterns of transcriptomic variation even in the presence of technical noise [36]. Likewise, integrative analyses combining cancer and normal RNA-seq data show that, after proper normalization, biological differences between conditions remain strongly associated with the dominant patterns of transcriptomic variation, while batch effects need to be addressed separately to distinguish biological from technical variation [37]. Multiple studies of large cancer cohorts have demonstrated clear separation between tumour and normal samples along the major principal components, with clustering patterns strongly associated with biological condition rather than technical batch effects. These findings further support the observation that biological grouping can represent a dominant pattern of variation captured by dimensionality reduction [38]. Furthermore, analyses of tumour and normal samples have shown that the first principal components are often associated with differences in gene expression profiles between biological conditions, while technical factors such as library size or sequencing batch may contribute to additional patterns of variation [39]. These published findings are consistent with our observation that biological status is associated with the dominant pattern of variation in cancer versus normal comparisons, whereas differences associated with the choice of reference transcriptome become more apparent when biological heterogeneity is reduced.

In the cancer-only comparison (Approach III), PCA revealed that variability was predominantly associated with the choice of reference transcriptome rather than with biological differences between samples, consistent with studies showing that differences in genome assemblies and their associated transcript annotations can introduce systematic shifts in expression estimates when the same RNA-seq data are reanalysed using different reference transcriptomes [40]. Differences between reference frameworks, including transcript structure revisions and the addition of novel isoforms, have been demonstrated to alter read assignment and abundance estimation, thereby generating measurable variability independent of biological signal [41]. Comparative evaluations of genome builds further confirm that annotation updates can modify the set of detected transcripts and influence downstream differential expression results, particularly in analyses focused on homogeneous sample groups where biological variance is limited [33]. Such annotation-dependent effects are especially pronounced in cancer transcriptomes due to extensive alternative splicing and transcript complexity, increasing sensitivity to reference definition changes [34]. Therefore, the choice of reference transcript may significantly impact gene expression analyses in breast cancer studies, underscoring recommendations that standardized and up-to-date genome references be adopted in clinical bioinformatics workflows to ensure reproducibility and comparability across laboratories [42].

In contrast, in the control-only comparison (Approach IV), variability associated with the choice of reference transcriptome was less dominant, while interindividual biological differences between samples were more apparent, consistent with observations that natural gene expression heterogeneity among individuals can constitute a major source of variance in healthy tissues [43]. Large-scale transcriptomic profiling of normal human tissues has demonstrated substantial interindividual variability driven by genetic and regulatory differences, which can exceed certain technical effects when biological diversity is present [44]. Together, these findings support our observation that reference transcriptome effects become most visible in biologically homogeneous datasets, whereas in more heterogeneous normal samples, intrinsic interindividual variation represents a stronger determinant of transcriptomic variance.

Reference framework differences represent an under-recognized source of bioinformatic batch effects in clinical genomics, as differences in gene models, transcript definitions, and exon boundaries between genome builds can significantly alter RNA-seq quantification and downstream differential expression results [33,40]. In cancer-only comparisons, reference sequence version can emerge as a dominant source of variability because transcriptome complexity and isoform diversity amplify the effects of reference-dependent read assignment and transcript abundance estimation [34,41]. Importantly, genes included in clinically relevant prognostic signatures and hereditary cancer panels showed relatively stable aggregate expression patterns under the analytical conditions investigated [23]. Nevertheless, specific biologically relevant genes such as *PTEN*, *RAD51D*, *NANOG*, and *SOX11* may exhibit reference-dependent expression discrepancies due to alternative splicing, transcript reclassification, or model updates, potentially influencing interpretation in precision oncology contexts [33,45].

Several of the genes whose rank shifted most between references have independent links to breast cancer, so the choice of reference can move clinically relevant loci and not only poorly annotated features. Two ranked higher under GRCh38: MDC1, which acts in the DNA-damage response and whose disruption sensitises lobular breast cancer to PARP inhibition [46], and RASSF5, part of a prognostic expression signature in triple-negative disease [47]. Three ranked higher under GRCh37: CDKN1C, which encodes the p57KIP2 tumour suppressor whose repression accompanies epithelial proliferation in breast tissue [48]; MSRA, part of a macrophage-related signature that predicts metastasis-free survival after adjuvant radiotherapy in triple-negative breast cancer [49]; and PLS3 (plastin-3), an established circulating-tumour-cell marker of long-term prognosis [50]. Because the rank of each gene depends on which reference transcriptome is used, an analysis that prioritises candidates by expression rank may reach a different conclusion about the same gene based on this choice alone.

The comparison of differential transcript expression between GRCh37 and GRCh38 annotations in MCF7 breast cancer and normal tissue samples reveals notable differences in gene profiling outcomes. Table 1 highlights outlier genes with significant expression changes depending on the reference transcriptome version used. Several genes, including *PTEN*, *RAD51D*, *MLLT1*, *TXNIP*, *PALM2-AKAP2*, and *HSPA14*, are consistently identified across both reference transcriptomes and are framed in black, indicating concordant differential expression between the two analytical approaches. Importantly, key cancer-related genes such as *PTEN* and *RAD51D*, involved in tumour suppression and DNA repair respectively, appear across multiple diagnostic panels and both conditions, underscoring their relevance in breast cancer biology. However, discrepancies also emerge: *NANOG*, a marker of pluripotency, and *SOX11*, linked to cancer progression, are found only in the case dataset, suggesting that transcriptome version can affect the detection of clinically significant genes.

Analysing the particular genes revealed by our research, summarised in Table 1, it is clear that several genes exhibit differential expression depending on the transcriptome used. For instance, *PTEN*, a critical tumour suppressor gene, shows such inconsistent expression. The discrepancies may arise from differences in transcript definitions between GRCh37 and GRCh38, affecting read pseudoalignment and quantification. The *PTEN* gene, located on chromosome 10q23.31, encodes a dual-specificity phosphatase that plays a pivotal role in regulating cell growth and survival [45,51]. Its primary function involves dephosphorylating phosphatidylinositol (3,4,5)-trisphosphate (PIP3), thereby antagonizing the PI3K/AKT signalling pathway, which is crucial for cell proliferation and survival [51]. Loss or mutation of *PTEN* leads to uncontrolled activation of the PI3K/AKT pathway, contributing to tumorigenesis in various cancers, including breast cancer. In breast cancer, *PTEN* loss is associated with increased tumour aggressiveness and poor prognosis [45]. Germline mutations in *PTEN* are linked to hereditary cancer syndromes such as Cowden syndrome, which elevates the risk for breast, thyroid, and endometrial cancers [52]. Beyond its tumour-suppressive functions, *PTEN* also plays a role in metabolic regulation, influencing processes like glycolysis and lipid metabolism. This dual role underscores the gene’s importance in maintaining cellular homeostasis. Moreover, *PTEN*’s involvement in DNA repair mechanisms, particularly in double-strand break repair, highlights its contribution to genomic stability. Deficiencies in *PTEN* can compromise DNA repair, leading to increased mutation rates and cancer progression. In summary, *PTEN* serves as a critical regulator of cellular functions, and its dysregulation has profound implications in cancer development and progression, making it a significant focus in oncology research and potential therapeutic interventions.

Similarly, *NANOG*, a gene associated with stemness and tumour progression, appears variably expressed depending on the reference transcritpme choice. This inconsistency can impact the assessment of tumour aggressiveness and potential treatment strategies [53]. *RAD51D*, involved in DNA repair, also shows annotation-dependent expression differences. Given its role in homologous recombination, accurate quantification is essential for evaluating genomic stability and potential responsiveness to PARP inhibitors [33].

An important finding was the relatively high stability of the evaluated gene expression captured in signatures and gene panels. The median expression of genes comprising the MammaPrint and Naderi signatures, as well as genes included in the evaluated hereditary cancer panels, did not differ significantly between GRCh37 and GRCh38 in the present analysis. This suggests that aggregate expression patterns across established gene sets may be less sensitive to reference transcriptome changes than individual genes. Nevertheless, these results should not be interpreted as demonstrating equivalence of the corresponding clinical assays or their clinical classifications. The present analysis assessed expression of the constituent genes rather than reproducing and independently validating the clinical algorithms used for patient risk stratification. In particular, MammaPrint is a clinically validated prognostic assay whose performance has been established in appropriately designed clinical cohorts [5,20,54]. Our findings therefore indicate relative stability of the analysed gene expression patterns across the two reference transcriptomes, rather than clinical validation of the corresponding prognostic classifications.

The variability in expression of these genes underscores the importance of selecting an appropriate and consistent reference transcriptome. Studies have shown that the choice of reference transcriptome can lead to significant differences in gene expression estimates, affecting downstream analyses and interpretations [33]. Moreover, the complexity of the reference transcriptome can influence the detection of differentially expressed genes and introduce ambiguity in read assignment, leading to increased variability in gene expression estimates [34]. In clinical settings, such discrepancies can have profound implications. For example, misclassification of tumour subtypes due to annotation-induced expression differences can lead to suboptimal treatment choices. Therefore, standardizing the reference framework used in clinical diagnostics is crucial. Recent advances in molecular profiling have facilitated the subclassification of TNBC into distinct molecular subtypes, each with unique genomic and transcriptomic landscapes. For instance, the FUTURE trial employed integrative analyses combining somatic mutations, copy number aberrations, and gene expression profiles to stratify TNBC patients into subtypes such as luminal androgen receptor (LAR), immunomodulatory (IM), basal-like immune-suppressed (BLIS), and mesenchymal-like (MES). Each subtype was associated with tailored therapeutic strategies, demonstrating the potential of precision medicine in improving outcomes for TNBC patients [55].

Our findings have implications for reproducibility and cross-study comparability in transcriptomic research. RNA-seq pipelines are often described primarily in terms of sequencing platform, alignment or quantification software, normalization method, and differential expression algorithm, whereas the exact reference transcriptome is sometimes reported less prominently. However, two laboratories using nominally similar analytical workflows may obtain different results if they use different genome builds or annotation releases. This problem may be particularly relevant when previously published datasets are reanalysed, when results from different institutions are integrated, or when exploratory biomarkers identified using one annotation are subsequently evaluated using another. A particularly relevant example is the extensive genomic data generated and analysed using GRCh37, including early TCGA studies, such as the comprehensive molecular characterization of breast cancer performed before GRCh38 became available [56]. The relevance of genome-build differences therefore extends beyond analytical pipelines that continue to use GRCh37 to previously published and publicly available datasets that remain widely used as reference points and are compared with more recent findings. Reanalysis of such datasets using GRCh38 could determine whether changes in genome assembly affect gene-level results and downstream biological interpretation. Direct comparison of the same sequencing data across genome builds could therefore provide valuable insight into the comparability of results generated using different reference versions. Accordingly, studies should explicitly document the genome build, annotation provider, transcriptome version, and associated processing parameters. Such version control is essential for reproducibility and meaningful comparison of RNA-seq results across studies.

The findings also highlight an important distinction between the stability of broad biological conclusions and the consistency of individual molecular measurements. In our dataset, the principal biological distinction between MCF-7 cells and normal breast tissue remained evident irrespective of annotation, and most enriched pathways were shared between the two approaches. At the same time, the identity and number of differentially expressed genes were not completely stable, and individual cancer-relevant genes could show substantial annotation-dependent differences. Therefore, the impact of reference transcriptome choice should not be evaluated solely in terms of whether the overall biological conclusions are preserved. For biomarker discovery and precision transcriptomics, the stability of individual genes, gene rankings, pathway scores, and clinically relevant molecular classifications may be equally important.

### Limitations of the Study

Several limitations should be considered when interpreting these findings. First, the study was based on a small publicly available dataset comprising two MCF7 breast cancer cell-line samples and two healthy breast tissue samples. Consequently, the statistical power to characterize biological variability and to generalize annotation-dependent effects across breast cancer populations is limited. In particular, the differential expression results should be regarded as exploratory rather than as definitive estimates of breast cancer-associated transcriptional changes.

Second, the cancer samples consisted of an established MCF7 cell line, whereas the control samples were derived from healthy breast tissue. This comparison therefore reflects not only differences related to malignant phenotype but also substantial differences in cellular composition, tissue context, and experimental environment. The observed cancer-versus-control separation should consequently not be interpreted as a direct representation of the transcriptional differences between primary breast tumours and normal breast tissue. Accordingly, the present study focuses on annotation-dependent variability rather than cancer-specific transcriptional changes.

Third, the present analysis evaluated only two reference transcriptomes, based on GRCh37 and GRCh38. Reference-dependent differences may also arise between transcriptome releases based on the same genome assembly and may vary depending on the annotation provider, transcript definitions, and the inclusion of coding and non-coding transcripts. Therefore, the present findings cannot be assumed to represent the full range of variability associated with different reference transcriptomes and reference frameworks.

Fourth, the analysis focused on a single transcript quantification framework based on Kallisto and a specific downstream differential expression workflow. Different quantification, transcript-to-gene summarization, normalization, and differential expression methods may respond differently to annotation changes. Thus, the magnitude and gene-level pattern of annotation-dependent variability observed here may not be identical across alternative bioinformatic pipelines.

Fifth, the case–case and control–control analyses compare alternative computational representations of the same underlying RNA-seq libraries rather than independent biological samples. These analyses were intended to characterize variability associated with the choice of reference transcriptome and should not be interpreted as conventional biological differential expression experiments. The statistical significance of individual genes in these comparisons therefore requires cautious interpretation, and effect-size and concordance-based measures may provide a more appropriate assessment of sensitivity to the choice of reference transcriptome.

Sixth, the assessment of MammaPrint, Naderi, and hereditary cancer gene panels was based on the expression of their constituent genes rather than on independent validation of the corresponding clinical assays or their validated scoring algorithms. The absence of significant differences in median expression across these gene sets therefore does not establish equivalence of clinical risk classification, diagnostic performance, or treatment recommendations between annotation versions.

Finally, the study did not include an independent breast cancer cohort for external validation, nor did it experimentally validate annotation-dependent differences in selected genes at the transcript or protein level. In particular, the clinical relevance of differences observed for genes such as *PTEN*, *RAD51D*, *NANOG*, *and SOX11* remains to be established. Future studies should therefore evaluate the reproducibility of these findings in larger, clinically annotated breast cancer cohorts, include multiple quantification methods, and determine whether annotation-dependent changes alter clinically relevant molecular classifications or biomarker scores.

Taken together, these limitations indicate that the present study should be considered a methodological proof-of-concept demonstrating measurable analytical variability associated with the choice of reference transcriptome. The findings support the need for explicit reporting of the reference transcriptome used and standardized computational workflows, while larger and independently validated cohorts are required to determine the extent to which such variability influences translational or clinical applications.

## 4. Material & Methods

### 4.1. Dataset

RNA-seq data from MCF7 breast cancer cells and healthy breast tissues were downloaded from the European Nucleotide Archive database (ENA). Data has been shared as a base of a project investigating the gene expression between normal and cancer cells (PRJEB4829). Data contains two controls (ERR358485, ERR358486) and two case samples of breast cancer (ERR358487, ERR358488). Control samples come from healthy breast tissues, and two case samples come from MCF7 breast cancer cell lines. Data was generated by Illumina HiSeq 2000 platform (Illumina, San Diego, CA, USA) in paired-end mode. The library size for each sample ranged from 73,974,766 to 97,983,949. However, the primary objective of this pilot study was not to characterize breast cancer-specific transcriptional alterations, but rather to assess the extent to which the choice of reference transcriptome can influence RNA-seq results and their interpretation. Two transcriptomes were taken from the Ensembl database: the recent one called GRCh38 (GRCh38.p13) and the previous one called GRCh37 (GRCh37.p13). All known transcripts, including both coding and non-coding, were used to encompass important features of cancer transcriptomics hidden not only directly in exons, but also in non-coding regions and allows for a comprehensive investigation of a much greater landscape of transcriptome expression changes.

### 4.2. Bioinformatics Workflow

#### Case–Control

The case–control pipeline consists of two bioinformatics approaches. Both are based on the same steps and software, but the difference is in the reference transcriptome used. One approach uses the transcriptome annotation based on the GRCh38 reference genome, while the second one uses the previous transcriptome annotation—GRCh37, which was last updated in 2015. The study aims to determine how the choice of reference transcriptome affects transcript expression estimates. The pipeline consisted of (i) quality control, (ii) quality-based data editing, (iii) expression quantification per transcript, and (iv) differential expression analysis. The quality control step (i) was done using FastQC v0.11.9 [57] and MultiQC v1.9 [58] programs. Quality-based data editing (ii) was performed using Trimmomatic v0.39 [59]. The options used were ILLUMINACLIP—to remove adaptors and other sequences specific for Illumina sequencer, SLIDINGWINDOW:4:20—to remove data with poor quality, and MINLEN:60—to drop out reads shorter than 60 bp. The first two steps were identical for all approaches used. The Kallisto software v0.46.2 [41] quantifies transcript expression (iii) based on the pseudoalignment process. The program was invoked twice: using GRCh37-based (Approach I) and GRCh38-based (Approach II) reference transcriptomes. At that point PCA plots were generated for expression of all genes, separately for Approach I and Approach II.

DESeq2 v1.50.2 [40] has been used to evaluate significant differences in transcript expression level (iv) between case (cancer) and control (normal) samples for Approach I and case and control samples for Approach II. DESeq2 is a widely established method for RNA-seq data that estimates gene-specific dispersion by borrowing information across genes, thereby providing more stable variance estimates in experiments with limited numbers of biological replicates [40]. Although a larger number of biological replicates would provide greater statistical power and more reliable estimation of biological variability, DESeq2 has been shown to perform well in RNA-seq experiments with limited replication, including with respect to false discovery rate control [40]. Given the small sample size of the present proof-of-concept study, the resulting differential expression analyses were therefore considered exploratory rather than definitive. Accordingly, identified DEGs were interpreted conservatively, with greater emphasis placed on concordance between annotation versions and on broader biological patterns than on individual statistically significant genes.

The agreement between Approaches I and II was further investigated using two complementary analyses. First, Spearman correlation test was used to assess the concordance of log2FC estimates obtained using the two reference transcriptomes. Correlations were calculated for all genes and, separately, for genes identified as statistically significant DEGs in both analyses. Second, gene-ranking concordance between Approaches I and II was assessed by comparing gene ranks derived from expression levels obtained using the two reference transcriptomes. Gene-ranking concordance was evaluated for all quantified genes and, separately, for genes identified as statistically significant DEGs in both analyses.

Additionally, for the results obtained from both approaches, all differentially expressed genes were subjected to enrichment analysis using gene sets from the Molecular Signatures Database (MSigDB) Hallmark collection, as well as pathway enrichment analysis based on the Kyoto Encyclopedia of Genes and Genomes (KEGG). The analysis was conducted separately for up- and down-regulated genes in order to capture directional changes.

### 4.3. Case-Case and Control-Control

**Figure 14:**
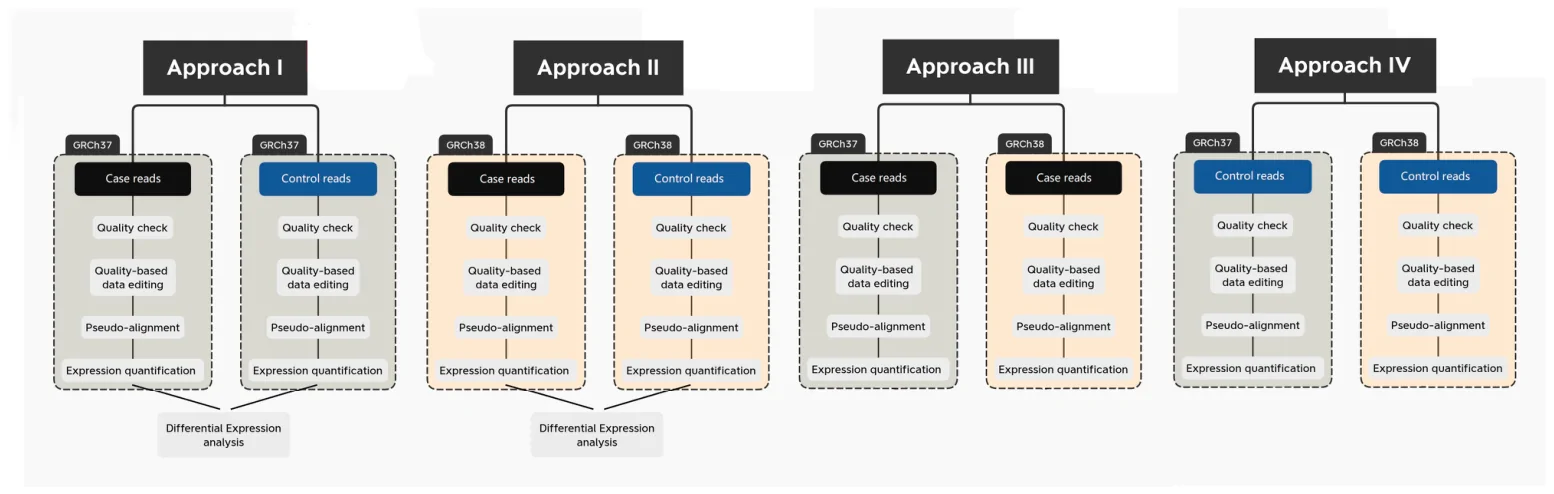
The diagram summarizes all analytical strategies implemented in the bioinformatic pipeline. In Approach I, gene expression in cancer and control samples was quantified using the GRCh37-based reference transcriptome, whereas in Approach II, quantification was performed using the GRCh38. Approach III comprised a case–case comparison of cancer samples quantified according to GRCh37 and GRCh38, while Approach IV involved an analogous control–control comparison between the two reference transcriptomes.

The two kallisto outputs containing quantified transcript expression data (iii) were subsequently used to identify genes whose estimated expression was affected by the choice of reference transcriptome (iv). However, this time case-case (Approach III) comparison was made in that way that cancer (case) samples were quantified based on the GRCh37-based transcriptome (the result of Kallisto in Approach I), while the other one based on GRCh38 (Approach II). Similarly, genes whose estimated expression was potentially affected by the choice of reference transcriptome were identified between the two control groups (Approach IV). Within Approach III, the concordance of raw gene expression estimates obtained using the GRCh37- and GRCh38-based reference transcriptomes was assessed using Spearman’s rank correlation, including calculation of the correlation coefficient (ρ) and evaluation of its statistical significance. PCA was performed for both Approaches. All Approaches are summarised in Figure 14.

#### Assessment of Clinically Relevant Gene Panels Across Genome Builds

**Figure 15:**
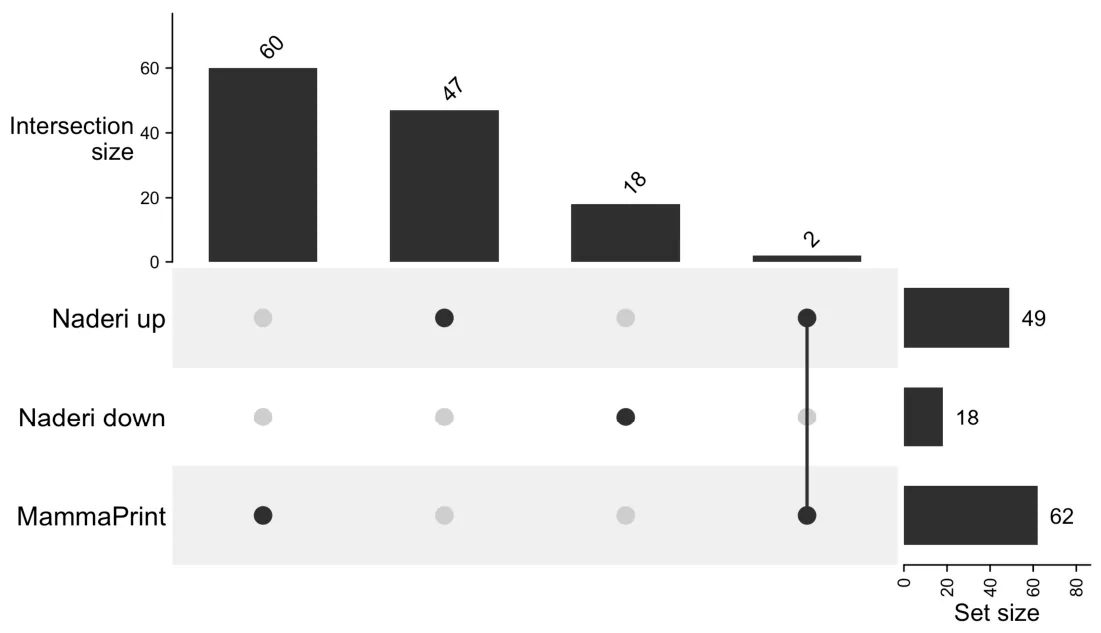
Overlap of Naderi and MammaPrint expression signatures.

**Figure 16:**
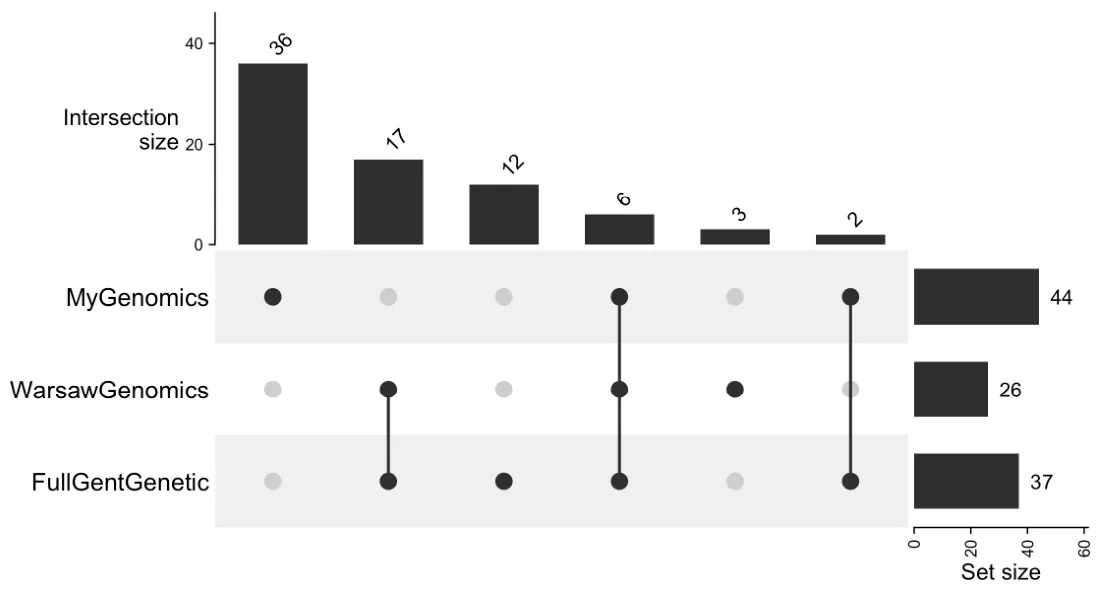
Overlap between commercially available gene panels: WarsawGenomics, FullGentGenetics, and MyGenomics.

One of the goals of this study was to investigate whether the choice between current and previous reference transcriptomes affects gene expression estimates and the interpretation of clinically relevant gene expression patterns. We compared expression prognostic signatures (set of genes proved to be correlated with the clinical outcome) between GRCh37-based and GRCh38-based reference transcriptomes to evaluate differences in the distribution of gene expression expressed by TPM (transcripts per million). (1) MammaPrint is a gene signature test that allows for the prediction of early-stage breast cancer and the assessment of distant metastasis risk in patients. It was determined through a complex process involving gene selection based on clinical outcomes, gene expression profiling (microarray technology), and validation in clinical studies, providing evidence that it can be used to personalize breast cancer treatment [5,20,21,22,54]. The original gene panel includes 70 genes, but the number of genes available to find in the literature is 66 [20,60,61]. (2) Moreover, expression signatures, for both upregulated (a) and downregulated (b) genes, significantly correlated with survival in breast cancer were defined by Naderi et al. [23]. These 70 prognostic genes were found using microarray expression profiling of 135 early-stage tumors. The comparison was also performed for genes from 3 diagnostics gene panels offered by the following companies: (3) WarsawGenomics (26 genes; www.warsawgenomics.pl), (4) FullGentGenetics (37 genes; www.fulgentgenetics.com) and (5) MyGenomics (45 genes; www.mygenomics.com). These three panels are devoted to determining the DNA variation in particular genes. The intention was to evaluate the expression behaviour of genes from established cancer-related panels as predefined transcriptomic gene sets, rather than to reproduce or validate their clinical applications. After expression quantification step done in this study, certain genes from the genomic panels were not present in our datasets. For example, genes such as LOC100288906, LOC730018, and LOC100131053 from Mammaprint were withdrawn by NCBI because their predicted models were not retained in a later annotation. Therefore, the final number of genes included in the analysis differed than the original number of genes characterising panels. The numbers of genes were: (1) 62 for Mammaprint, (2a) 49 for Naderi up- and (2b) 18 for downregulated genes, (3) 26 for WarsawGenomics, (4) 37 for FullGentGenetics and (5) 44 for MyGenomics. The full list of genes is provided in Supplementary Material S3. The overlap between expression signatures (Figure 15) and gene panels (Figure 16) were visualised on Upset plots.

The comparison of gene expression, due to lack of normality, was performed using the Wilcoxon signed rank test. The null hypothesis assumed that the median of the differences in expression levels for genes quantified using the GRCh37-based and GRCh38-based transcriptomes was equal to zero. Statistical analyses were performed for both genes included in the expression signature and gene panels. Genes exhibiting significant differences in expression between GRCh37 and GRCh38 were determined based on bootstrap percentile confidence intervals (10,000 bootstrap samples). A gene was considered significantly differentially expressed if the difference in its expression between annotations lay outside the 99% confidence interval constructed from the distribution of expression differences across all genes. To enable comparative analysis, the R package was utilized for computational analyses and graphical visualizations (dplyr, tidyr, ggplot2, gridExtra) [62].

## 5. Conclusions

Our pilot study demonstrates that the choice of reference transcriptome constitutes a measurable and non-negligible source of variability in RNA-seq-based differential gene expression analyses in breast cancer. By systematically comparing GRCh37- and GRCh38-based reference transcriptomes using identical bioinformatic workflows, we show that although the global separation between cancer and normal samples is largely consistent across the two analyses, the number, identity, and ranking of differentially expressed genes vary with the choice of reference transcriptome. These differences stem from changes in transcript definitions, exon structures, and the inclusion of newly annotated coding and non-coding transcripts, which directly affect read assignment and transcript quantification.

Importantly, our findings indicate that established clinical gene expression signatures and commonly used diagnostic gene panels are largely stable across reference transcripts. Median expression levels of genes included in prognostic tools such as MammaPrint and Naderi signatures, as well as selected diagnostic panels, did not differ significantly between GRCh37 and GRCh38. This suggests that well-validated, aggregate gene sets used in clinical decision-making may be relatively resilient to transcriptome updates, supporting their continued applicability across bioinformatic environments.

However, the analysis also reveals that specific biologically and clinically relevant genes, including key regulators involved in tumour suppression, DNA repair, and stemness, can display annotation-dependent discrepancies in expression estimates. In the comparison of cancer samples quantified using different reference transcriptomes, variability associated with the choice of reference transcriptome represented a major component of the observed variation. However, this observation may be specific to the dataset analysed and should not necessarily be generalised to breast cancer transcriptomic studies more broadly. Nevertheless, these findings highlight the importance of considering choice of reference transcriptome as a potential source of technical variability when interpreting gene expression differences.

Collectively, our results highlight the transcriptome reference as a “hidden technical variable” in RNA-seq studies. While major biological conclusions may remain stable, transcriptome choice can influence fine-scale interpretation and reproducibility. Therefore, standardization toward updated and explicitly reported reference, along with periodic reanalysis of previously generated datasets, is essential to ensure methodological transparency and comparability across studies. As precision oncology increasingly relies on high-resolution molecular profiling, careful consideration of reference genome and transcriptome builds should become an integral component of best practices in clinical bioinformatics.

## Figures and Tables

**Table 1:** Presents outlier genes exhibiting significant differences in expression estimates between the GRCh37- and GRCh38-based reference transcriptomes. Genes shared between panels are highlighted with a black frame, while those common between case and control samples are shown in bold italics.

Case					
**MammaPrint**	**Naderi up**	**Naderi down**	**WarsawGenomics**	**FullGentGenetics**	**MyGenomics**
LPCAT1	***HSPA14***	***NANOG***	***PTEN***	***PTEN***	PTEN
***PALM2-AKAP2***	***MLLT1***	***TXNIP***	***RAD51D***	***RAD51D***	
***RASSF7***	***PMCH***		***PMS2***	FAM175A	
***SERF1A***	SOX11			***MRE11A***	
**Control**					
**MammaPrint**	**Naderi up**	**Naderi down**	**WarsawGenomics**	**FullGentGenetics**	**MyGenomics**
***PALM2-AKAP2***	***HSPA14***	***NANOG***	***PTEN***	PTEN	PTEN
***RASSF7***	***MLLT1***	***TXNIP***	***RAD51D***	***RAD51D***	
***SERF1A***	***PMCH***		***PMS2***	RECQL	
	RNFT2			***MRE11A***	

## Data Availability

The transcriptomic data (RNA-seq) were obtained from the European Nucleotide Archive database and can be found under PRJEB4829 project ID. Transcriptome annotations were taken from the Ensemble database including the most recent called GRCh38 and the previous one called GRCh37.

## Supplementary Materials

The following supporting information can be downloaded at: https://www.mdpi.com/article/10.3390/ijms27219398/s1.

## Abbreviations

BLIS	basal-like immune-suppressed subtype
cDNA	complementary deoxyribonucleic acid
DEG	differentially expressed genes
ENA	European Nucleotide Archive
GRCh37	Genome Reference Consortium human build 37
GRCh38	Genome Reference Consortium human build 38
IM	immunomodulatory subtype
KEGG	Kyoto Encyclopedia of Genes and Genomes
LAR	luminal androgen receptor subtype
MCF7	Michigan Cancer Foundation-7
MES	mesenchymal-like subtype
MSigDB	Molecular Signatures Database
ncRNA	non-coding ribonucleic acid
NGS	next-generation sequencing
PCA	principal component analysis
PC1	first principal component
TCGA	The Cancer Genome Atlas
TNBC	triple-negative breast cancer
TPM	transcripts per million

## References

[1] Sung H., Ferlay J., Siegel R.L., Laversanne M., Soerjomataram I., Jemal A., Bray F. (2021). Global Cancer Statistics 2020: GLOBOCAN Estimates of Incidence and Mortality Worldwide for 36 Cancers in 185 Countries. CA Cancer J. Clin..

[2] Watkins E.J. (2019). Overview of breast cancer. JAAPA.

[3] Gadaleta E., Thorn G.J., Ross-Adams H., Jones L.J., Chelala C. (2022). Field cancerization in breast cancer. J. Pathol..

[4] Comşa Ş., Cîmpean A.M., Raica M. (2015). The Story of MCF-7 Breast Cancer Cell Line: 40 Years of Experience in Research. Anticancer Res..

[5] Council Health Quality Ontario (2020). Gene expression profiling tests for early-stage invasive breast cancer: A health technology assessment. Ont. Health Technol. Assess. Ser..

[6] Desmedt C., Voet T., Sotiriou C., Campbell P.J. (2012). Next-generation sequencing in breast cancer: First take home messages. Curr. Opin. Oncol..

[7] Guillen K.P., Fujita M., Butterfield A.J., Scherer S.D., Bailey M.H., Chu Z., DeRose Y.S., Zhao L., Cortes-Sanchez E., Yang C.-H. (2022). A human breast cancer-derived xenograft and organoid platform for drug discovery and precision oncology. Nat. Cancer.

[8] Nourieh M., Vibert R., Saint-Ghislain M., Cyrta J., Vincent-Salomon A. (2023). Next-generation sequencing in breast pathology: Real impact on routine practice over a decade since its introduction. Histopathology.

[9] Akcakanat A., Zheng X., Pico C.X.C., Kim T.-B., Chen K., Korkut A., Sahin A., Holla V., Tarco E., Singh G. (2021). Genomic, Transcriptomic, and Proteomic Profiling of Metastatic Breast Cancer. Clin. Cancer Res..

[10] Bao T., Davidson N.E. (2008). Gene expression profiling of breast cancer. Adv. Surg..

[11] Bao Y., Wang L., Shi L., Yun F., Liu X., Chen Y., Chen C., Ren Y., Jia Y. (2019). Transcriptome profiling revealed multiple genes and ECM-receptor interaction pathways that may be associated with breast cancer. Cell. Mol. Biol. Lett..

[12] Lu P., Foley J., Zhu C., McNamara K., Sirinukunwattana K., Vennam S., Varma S., Fehri H., Srivastava A., Zhu S. (2021). Transcriptome and genome evolution during HER2-amplified breast neoplasia. Breast Cancer Res..

[13] Yin Y., Zhang J., Li X., Duan M., Zhao M., Zhang F., Chamba Y., Shang P. (2024). Application of RNA-Seq Technology for Screening Reproduction-Related Differentially Expressed Genes in Tibetan and Yorkshire Pig Ovarian Tissue. Vet. Sci..

[14] Cunningham F., Allen J.E., Allen J., Alvarez-Jarreta J., Amode M.R., Armean I.M., Austine-Orimoloye O., Azov A.G., Barnes I., Bennett R. (2022). Ensembl 2022. Nucleic Acids Res..

[15] Chen H., Fan S., Stone J., Thompson D.J., Douglas J., Li S., Scott C., Bolla M.K., Wang Q., Dennis J. (2022). Genome-wide and transcriptome-wide association studies of mammographic density phenotypes reveal novel loci. Breast Cancer Res..

[16] Hakkaart C., Pearson J.F., Marquart L., Dennis J., Wiggins G.A.R., Barnes D.R., Robinson B.A., Mace P.D., Aittomäki K., Andrulis I.L. (2022). Copy number variants as modifiers of breast cancer risk for BRCA1/BRCA2 pathogenic variant carriers. Commun. Biol..

[17] Tsaousis G.N., Papadopoulou E., Agiannitopoulos K., Pepe G., Tsoulos N., Boukovinas I., Floros T., Iosifidou R., Katopodi O., Koumarianou A. (2022). Revisiting the Implications of Positive Germline Testing Results Using Multi-Gene Panels in Breast Cancer Patients. Cancer Genom. Proteom..

[18] Liberzon A., Birger C., Thorvaldsdóttir H., Ghandi M., Mesirov J.P., Tamayo P. (2015). The Molecular Signatures Database Hallmark Gene Set Collection. Cell Syst..

[19] Kanehisa M., Furumichi M., Sato Y., Matsuura Y., Ishiguro-Watanabe M. (2025). KEGG: Biological systems database as a model of the real world. Nucleic Acids Res..

[20] Haan J.C., Bhaskaran R., Ellappalayam A., Bijl Y., Griffioen C.J., Lujinovic E., Audeh W.M., Penault-Llorca F., Mittempergher L., Glas A.M. (2022). MammaPrint and BluePrint comprehensively capture the cancer hallmarks in early-stage breast cancer patients. Genes Chromosomes Cancer.

[21] Cardoso A.R., Oliveira M., Amorim A., Azevedo L. (2016). Major influence of repetitive elements on disease-associated copy number variants (CNVs). Hum. Genom..

[22] Wittner B.S., Sgroi D.C., Ryan P.D., Bruinsma T.J., Glas A.M., Male A., Dahiya S., Habin K., Bernards R., Haber D.A. (2008). Analysis of the MammaPrint breast cancer assay in a predominantly postmenopausal cohort. Clin. Cancer Res..

[23] Naderi A., Teschendorff A.E., Barbosa-Morais N.L., Pinder S.E., Green A.R., Powe D.G., Robertson J.F.R., Aparicio S., Ellis I.O., Brenton J.D. (2007). A gene-expression signature to predict survival in breast cancer across independent data sets. Oncogene.

[24] Bhushan A., Gonsalves A., Menon J.U. (2021). Current State of Breast Cancer Diagnosis, Treatment, and Theranostics. Pharmaceutics.

[25] He Z., Chen Z., Tan M., Elingarami S., Liu Y., Li T., Deng Y., He N., Li S., Fu J. (2020). A review on methods for diagnosis of breast cancer cells and tissues. Cell Prolif..

[26] Aminolroayaei F., Shahbazi-Gahrouei S., Khorasani A., Shahbazi-Gahrouei D. (2024). A Review of Imaging Methods and Recent Nanoparticles for Breast Cancer Diagnosis. Information.

[27] Tjota M.Y., Segal J.P., Wang P. (2024). Clinical Utility and Benefits of Comprehensive Genomic Profiling in Cancer. J. Appl. Lab. Med..

[28] Berger M.F., Mardis E.R. (2018). The emerging clinical relevance of genomics in cancer medicine. Nat. Rev. Clin. Oncol..

[29] Juan C., Peng Y. (2025). The Molecular Landscape and Utility of Multiomic Analyses in Triple-Negative Breast Cancer: Further Subtyping and Exploring Novel Biomarkers and Therapeutic Targets. Cancers.

[30] Meric-Bernstam F., Makker V., Oaknin A., Oh D.-Y., Banerjee S., González-Martín A., Jung K.H., Ługowska I., Manso L., Manzano A. (2024). Efficacy and Safety of Trastuzumab Deruxtecan in Patients with HER2-Expressing Solid Tumors: Primary Results from the DESTINY-PanTumor02 Phase II Trial. J. Clin. Oncol..

[31] Shao Q., Deng J., Wu H., Huang Z. (2025). HER2-positive gastric cancer: From targeted therapy to CAR-T cell therapy. Front. Immunol..

[32] Drilon A., Laetsch T.W., Kummar S., DuBois S.G., Lassen U.N., Demetri G.D., Nathenson M., Doebele R.C., Farago A.F., Pappo A.S. (2018). Efficacy of Larotrectinib in TRK Fusion-Positive Cancers in Adults and Children. N. Engl. J. Med..

[33] Chisanga D., Liao Y., Shi W. (2022). Impact of gene annotation choice on the quantification of RNA-seq data. BMC Bioinform..

[34] Hamaguchi Y., Zeng C., Hamada M. (2021). Impact of human gene annotations on RNA-seq differential expression analysis. BMC Genom..

[35] Gonzalez A., Leon D.A., Perera Y., Perez R. (2023). On the gene expression landscape of cancer. PLoS ONE.

[36] Arora S., Pattwell S.S., Holland E.C., Bolouri H. (2020). Variability in estimated gene expression among commonly used RNA-seq pipelines. Sci. Rep..

[37] Wang Q., Armenia J., Zhang C., Penson A.V., Reznik E., Zhang L., Minet T., Ochoa A., Gross B.E., Iacobuzio-Donahue C.A. (2018). Unifying cancer and normal RNA sequencing data from different sources. Sci. Data.

[38] Perera Y., Gonzalez A., Perez R. (2021). Principal component analysis of RNA-seq data unveils a novel prostate cancer-associated gene expression signature. bioRxiv.

[39] Molania R., Foroutan M., Gagnon-Bartsch J.A., Gandolfo L.C., Jain A., Sinha A., Olshansky G., Dobrovic A., Papenfuss A.T., Speed T.P. (2023). Removing unwanted variation from large-scale RNA sequencing data with PRPS. Nat. Biotechnol..

[40] Love M.I., Huber W., Anders S. (2014). Moderated estimation of fold change and dispersion for RNA-seq data with DESeq2. Genome Biol..

[41] Bray N.L., Pimentel H., Melsted P., Pachter L. (2016). Near-optimal probabilistic RNA-seq quantification. Nat. Biotechnol..

[42] Noman AAl Saba AAl Sayem M., Yasmin T., Nabi A.H.M.N. (2025). Identification of potential hub genes and molecular mechanisms in breast cancer microenvironment: A comprehensive transcriptomics approach. Medicine.

[43] Human Genomics (2015). The Genotype-Tissue Expression (GTEx) pilot analysis: Multitissue gene regulation in humans. Science.

[44] GTEx Consortium (2017). Genetic effects on gene expression across human tissues. Nature.

[45] Sajjadi E., Venetis K., Piciotti R., Gambini D., Blundo C., Runza L., Ferrero S., Guerini-Rocco E., Fusco N. (2021). Combined analysis of PTEN, HER2, and hormone receptors status: Remodeling breast cancer risk profiling. BMC Cancer.

[46] Sottnik J.L., Shackleford M.T., Nesiba C.S., Richer A.L., Fleischmann Z., Swartz J.M., Rowland C.E., Musick M., Fu R., Myler L.R. (2026). Altered MDC1 Interactions and Dysfunctional DNA Repair in Lobular Breast Cancer Confers Sensitivity to PARP Inhibition. Cancer Res..

[47] Sato K., Miura K., Tamori S., Akimoto K. (2024). Identification of a Gene Expression Signature to Predict the Risk of Early Recurrence and the Degree of Immune Cell Infiltration in Triple-Negative Breast Cancer. Cancer Genom. Proteom..

[48] Ogony J.W., Pacheco-Spann L.M., Arnold A., Cabezas J.V., Cruz-Reyes N., Erak C.P., Westerman P.J., Touré S.A., Akhtar S., Winham S.J. (2025). A STAT1-GBP1 axis modulates epithelial proliferation in postpartum breast tissue by repressing CDKI expression. Breast Cancer Res..

[49] Ye Y., Ma J., Zhang Q., Xiong K., Zhang Z., Chen C., Xiao H., Wang D. (2021). A CTL/M2 macrophage-related four-gene signature predicting metastasis-free survival in triple-negative breast cancer treated with adjuvant radiotherapy. Breast Cancer Res. Treat..

[50] Ueo H., Sugimachi K., Gorges T.M., Bartkowiak K., Yokobori T., Müller V., Shinden Y., Ueda M., Mori M., Kuwano H. (2015). Circulating tumour cell-derived plastin3 is a novel marker for predicting long-term prognosis in patients with breast cancer. Br. J. Cancer.

[51] Wang X., Huang H., Young K.H. (2015). The PTEN tumor suppressor gene and its role in lymphoma pathogenesis. Aging.

[52] Hendricks L.A.J., Hoogerbrugge N., Schuurs-Hoeijmakers J.H.M., Vos J.R. (2021). A review on age-related cancer risks in PTEN hamartoma tumor syndrome. Clin. Genet..

[53] Wu P.-Y., Phan J.H., Wang M.D. (2013). Assessing the impact of human genome annotation choice on RNA-seq expression estimates. BMC Bioinform..

[54] Mansani F.P., Freitas-Junior R. (2023). Validation of the 70-gene signature test (MammaPrint) to identify patients with breast cancer aged ≥70 years with ultralow risk of distant recurrence: A population-based cohort study. J. Geriatr. Oncol..

[55] Hossain F., Majumder S., David J., Miele L. (2021). Precision Medicine and Triple-Negative Breast Cancer: Current Landscape and Future Directions. Cancers.

[56] The Cancer Genome Atlas Network (2012). Comprehensive molecular portraits of human breast tumours. Nature.

[57] Andrews S. (2010). FastQC: A Quality Control Tool for High Throughput Sequence Data.

[58] Ewels P., Magnusson M., Lundin S., Käller M. (2016). MultiQC: Summarize analysis results for multiple tools and samples in a single report. Bioinformatics.

[59] Bolger A.M., Lohse M., Usadel B. (2014). Trimmomatic: A flexible trimmer for Illumina sequence data. Bioinformatics.

[60] Mosly D., Turnbull A., Sims A., Ward C., Langdon S. (2018). Predictive markers of endocrine response in breast cancer. World J. Exp. Med..

[61] Tian S., Roepman P., Van’t Veer L.J., Bernards R., de Snoo F., Glas A.M. (2010). Biological functions of the genes in the mammaprint breast cancer profile reflect the hallmarks of cancer. Biomark. Insights.

[62] R Core Team (2013). R: A Language and Environment for Statistical Computing.

